# A human immunodeficiency syndrome caused by mutations in *CARMIL2*

**DOI:** 10.1038/ncomms14209

**Published:** 2017-01-23

**Authors:** T. Schober, T. Magg, M. Laschinger, M. Rohlfs, N. D. Linhares, J. Puchalka, T. Weisser, K. Fehlner, J. Mautner, C. Walz, K. Hussein, G. Jaeger, B. Kammer, I. Schmid, M. Bahia, S. D. Pena, U. Behrends, B. H. Belohradsky, C. Klein, F. Hauck

**Affiliations:** 1 Dr. von Hauner Children's Hospital, Ludwig-Maximilians-Universität (LMU), Lindwurmstrasse 4, D-80337 Munich, Germany; 2 Department of Surgery, Technische Universität München (TUM), Ismaninger Strasse 22, D-81675 Munich, Germany; 3 Laboratory of Clinical Genomics, Federal University of Minas Gerais, 190 Professor Alfredo Balena Avenida, Belo Horizonte 30130-100, Brazil; 4 Research Unit Gene Vectors, Helmholtz Zentrum München (HMGU)–German Research Center for Environmental Health, Marchioninistrasse 25, D-81377 Munich, Germany; 5 Children's Hospital, Technische Universität München (TUM), Munich D-80804, Germany; 6 German Centre for Infection Research (DZIF), Trogerstrasse 30, D-81675 Munich, Germany; 7 Institute of Pathology, Ludwig-Maximilians-Universität (LMU), Thalkirchner Strasse 36, D-80337 Munich, Germany; 8 Institute of Pathology, Hannover Medical School (MHH), Carl-Neuberg-Strasse 1, D-30625 Hanover, Germany; 9 Department of Diagnostic Virology, Max von Pettenkofer-Institute, Ludwig-Maximilians-Universität (LMU), Pettenkoferstrasse 9a, D-80336 Munich, Germany; 10 Department of Pediatric Gastroenterology, Federal University of Minas Gerais, 110 Prof. Alfredo Balena Avenida, Belo Horizonte 30130-100, Brazil

## Abstract

Human T-cell function is dependent on T-cell antigen receptor (TCR) and co-signalling as evidenced by immunodeficiencies affecting TCR-dependent signalling pathways. Here, we show four human patients with EBV^+^ disseminated smooth muscle tumours that carry two homozygous loss-of-function mutations in the *CARMIL2* (*RLTPR*) gene encoding the capping protein regulator and myosin 1 linker 2. These patients lack regulatory T cells without evidence of organ-specific autoimmunity, and have defective CD28 co-signalling associated with impaired T-cell activation, differentiation and function, as well as perturbed cytoskeletal organization associated with T-cell polarity and migration disorders. Human CARMIL2-deficiency is therefore an autosomal recessive primary immunodeficiency disorder associated with defective CD28-mediated TCR co-signalling and impaired cytoskeletal dynamics.

## Abstract (web summary)

CARMIL2 (Rltpr) is involved in T-cell function. Here, the authors identify human CARMIL2-deficiency as an autosomal recessive primary immunodeficiency disorder characterized by EBV^+^ smooth muscle tumours, CD28 co-signalling deficiency and impaired cytoskeletal dynamics.

Studying primary immunodeficiency disorders (PID) has provided fundamental insights into principles governing human tolerance and immunity^1^. Co-signalling disorders such as CD40LG, SAP, OX40 or CD27-deficiency have highlighted unique properties of human host defence^2^. These disorders impair immune cell interactions and are associated with selective susceptibility to infectious agents^3^,^4^,^5^, but also shed light on the critical role of anti-tumour immune surveillance. In particular, insufficient T-cell immunity against human gamma herpesvirus-infected cells can lead to malignancy^6^. Whereas SAP-deficiency and CD27-deficiency predispose specifically to Epstein–Barr virus (EBV) infection and EBV-induced lymphoproliferative disorders, OX40-deficiency is associated with Kaposi's sarcoma induced by Kaposi's sarcoma herpesvirus^3^,^7^.

T cells are central in adaptive immunity, and T-cell signalling and co-signalling govern immune defence, immune homoeostasis and immune surveillance. On antigen encounter, the T-cell antigen receptor (TCR) becomes engaged and in concert with context-sensitive co-receptors, such as CD28, ICOS and OX40, induces T-cell activation, differentiation and function^8^,^9^. Although TCR-dependent signalling is one of the most studied signalling cascades, knowledge of CD28 co-signalling is incomplete^8^,^9^.

In a murine *N*-ethyl-*N*-nitrosourea-mutagenesis screen, the lymphoid lineage-specific actin-uncapping protein Rltpr (RGD motif, leucine-rich repeats, tropomodulin domain and proline-rich containing; murine homologue to human CARMIL2) was identified to be essential for CD28 co-signalling and regulatory T (T_reg_) cell development^10^. Mice expressing mutated Rltpr were not able to transduce CD28 co-signalling to its effector protein kinase c theta (Pkcθ). Consequently, *Rltpr*-mutant mice were specifically and completely devoid of T_reg_ and thus functionally phenocopied *Cd28*^−/−^ mice^10^. Furthermore, truncated Rltpr expression was associated with increased antibody-dependent CD28 internalization probably reflecting deregulated cytoskeletal dynamics^10^.

Human *CARMIL2* (*RLTPR*) was first described as a gene downregulated in cutaneous lesions of patients with psoriasis vulgaris^11^. The *CARMIL* family includes three human paralogs: *CARMIL1* (*LRRC16A*), *CARMIL2* (*LRRC16C*) and *CARMIL3* (*LRRC16B*). All proteins have a common domain architecture, that is, one N-terminal pleckstrin homology (PH) domain, several leucine-rich repeats (LRR), one capping protein binding region (CBR) domain and one C-terminal proline-rich domain (PRD)^11^,^12^. The CBR domain reduces the affinity of CP for actin filament barbed ends and makes them accessible for further actin polymerisation^13^. In contrast to CARMIL1 and CARMIL3, CARMIL2 orchestrates cell polarity by modulating microtubules and intermediate filaments^12^.

In this study, we describe homozygous loss-of-function *CARMIL2* mutations in four human patients from two different families presenting with the highly unusual phenotype of disseminated EBV^+^ smooth muscle tumours (SMT). Our phenotypic and functional studies demonstrate defective CD28 co-signalling with impaired T-cell activation, differentiation and function, and defective cytoskeletal organization associated with T-cell polarity and migration disorders.

## Results

### Case reports

**Figure 1:**
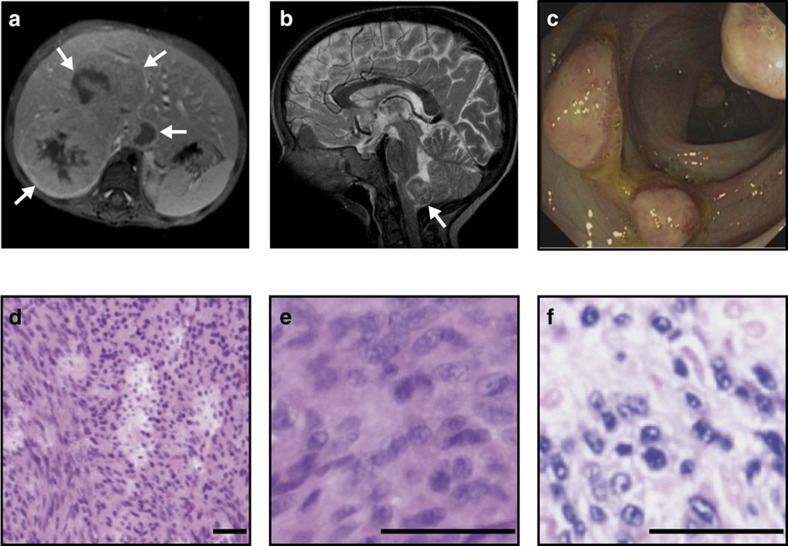
Disseminated EBV^+^ SMT in CARMIL2-deficient patients. (**a**) Abdominal magnetic resonance (MRI; T1 fat-sat post contrast medium) image of P1.2 with a tumour of ∼6 cm diameter in liver segments I and V–VIII (white arrows). (**b**) Cranial MRI (T2-WI) of P1.2 with a tumour of ∼1.7 cm diameter in the dorsal medulla oblongata (white arrow). (**c**) Colonoscopy image of P1.2 with multiple protruding tumors in the colon. (**d**,**e**) P2.1 hematoxylin and eosin (H&E) stains with leiomyogenic tumour cells and (**f**) EBER *in situ* hybridization. Scale bars, 50 μm. MRI, colonoscopy, tumour histopathology and EBER stains have been performed for four patients (P1.1, P1.2, P2.1 and P2.2).

Patients P1.1 and P1.2 were born from a consanguineous Yemenite couple. At the age of 2 years, P1.2 presented with severe failure to thrive, chronic diarrhoea, recurrent skin and upper airway infections (Table 1). At age 4, P1.2 was diagnosed with an EBV^+^ latency type III SMT in the liver (Fig. 1, Supplementary Fig. 1 and Supplementary Table 1). Tumour staging revealed additional 30 EBV^+^ SMT in the colon, terminal ileum and medulla oblongata (Fig. 1 and Table 1). Liver and brain EBV^+^ SMT were excised and the patient was treated by an individualized chemotherapy regimen with oral cyclophosphamide. However, disease progressed with additional lesions appearing in the brain, liver and spleen. Considering a therapy-refractory course, the family moved back to Yemen where the patient deceased.

At the age of 7 years, P1.1 presented with failure to thrive, recurrent upper airway infections, eczematous dermatitis and skin warts. Colonoscopy and histopathology revealed several EBV^+^ SMT (Table 1 and Supplementary Fig. 1). Further tumour staging and treatment were hindered by the family's decision to return to Yemen. Five years later, P1.1 is reported to have progressive disease.

**Figure 2:**
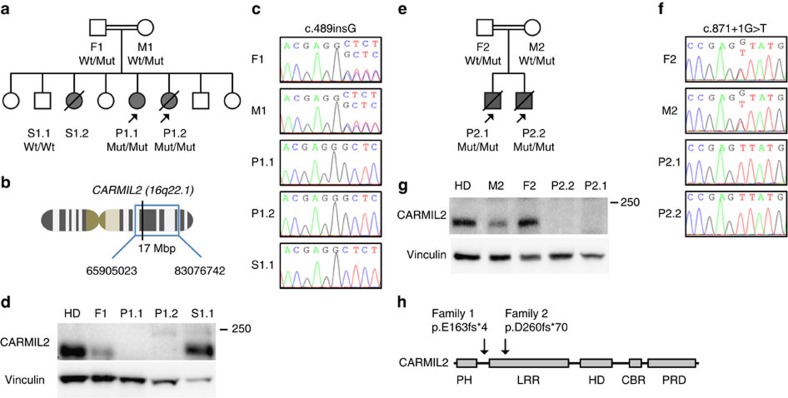
Homozygous *CARMIL2* mutations segregate with the disease phenotype. (**a**) Pedigree of family 1 with father (F1), mother (M1), siblings (S1.1 and S1.2) and patients (P1.1 and P1.2). Grey symbols and diagonal bars indicate diseased and deceased subjects, respectively, and *CARMIL2* wildtype (Wt) and *CARMIL2* c.489insG mutated (Mut) alleles are depicted for each patient. (**b**) Schematic representation of chromosome 16, cytogenetic band 16q22.1 and the homozygous region (blue box and bp interval) identified by SNP chip that harbour *CARMIL2* (black vertical line). (**c**) Electropherograms of family 1 members for *CARMIL2* Wt/Mut, Mut/Mut and Wt/Wt alleles. (**d**) CARMIL2 immunoblots of healthy donor (HD) and family 1 members with vinculin loading control. (**e**) Pedigree of family 2 with father (F2), mother (M2) and patients (P2.1 and P2.2). (**f**) Electropherograms of family 2 members for *CARMIL2* Wt/Mut and Mut/Mut. (**g**) CARMIL2 immunoblot of HD and family 2 members with vinculin loading control. (**h**) Schematic representation of CARMIL2 protein domain architecture and localization of family 1 p.E163fs*4 and family 2 p.D260fs*70 mutations. Immunoblots in (**d**) and (**g**) have been repeated three times.

Family 1 has six additional children and sibling 2 (S1.2) reportedly had died at age 19 in Yemen from a similar disease while the other siblings are healthy (Fig. 2a).

P2.1 and P2.2 were born from a consanguineous Brazilian couple and have been reported as cases of familial infantile myofibromatosis (Fig. 2e)^14^. At the age of 1 year, P2.1 developed chronic diarrhoea and, from age 4 on, repetitive infections with *Giardia* spp., pneumonias and skin warts (Table 1). At age 8, failure to thrive was documented and consecutively multiple gastrointestinal, pulmonary and a solid liver EBV^+^ SMT were detected (Table 1). P.2.1 received antineoplastic treatment with methotrexate and vinblastine, but disease progressed and he deceased at age 14.

P2.2 presented with recurrent chronic eczematous dermatitis from the age of 1 year on. At age 5 and 6, he had pneumonia and chickenpox, respectively. At age 6, failure to thrive and multiple liver and gastrointestinal EBV^+^ SMT were documented. At age 11, he had a second episode of chickenpox (Table 1). Despite antineoplastic treatment with methotrexate and vinblastine, the disseminated soft tissue tumours were progressing and he deceased at age 12.

In summary, the patients presented with chronic diarrhoea and failure to thrive, EBV^+^ SMT, recurrent viral and parasitic infections and dermatitis (Fig. 1, Supplementary Fig. 1 and Table 1).

### Immune phenotype and T-cell EBV-response

**Figure 3:**
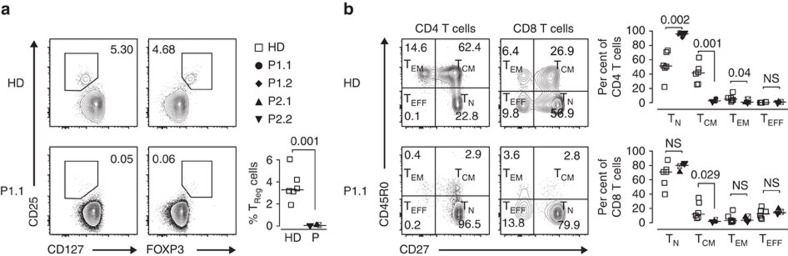
CARMIL2-deficiency impairs CD28-mediated T-cell differentiation. (**a**) Contour plots of CD25^+^CD127^low^ and CD25^+^FOXP3^+^ T_reg_ and summary of T_reg_ percentages for six HD (open squares), P1.1 (black circle), P1.2 (black rhomb), P2.1 (black up-pointing triangle) and P2.2 (black down-pointing triangle). (**b**) Contour plots of CD4 and CD8 naive (T_N_, CD45R0^−^CD27^+^), central memory (T_CM_, CD45R0^+^CD27^+^), effector memory (T_EM_, CD45R0^+^CD27^−^) and effector (T_EFF_, CD45R0^−^CD27^−^) T-cells (corresponding percentages are indicated in each square) and summary of CD4 and CD8 T-cell subtype percentages for six HD and four patients. Small horizontal lines indicate the median. Each symbol represents an individual donor. Data are representative for four independent experiments with *n*=2. Significance levels are calculated with Welch's *t*-test and indicated in the summary graphs (NS=non significant).

As the patients' clinical course and EBV^+^ SMT pointed towards defective T-cell immunity, we performed immune phenotyping (Supplementary Tables 2 and 3)^15^. Total peripheral T, B and NK cell counts mostly were within normal limits (Supplementary Tables 2b and 3b), but there was a profound reduction of T_reg_ (Fig. 3a). P2.1 and P2.2 had increased recent thymic emigrants (RTE) (Supplementary Table 3d) and all patients had a significant increase in naive helper T cells (CD4 T_N_) and insufficient gain of central memory helper T cells (CD4 T_CM_) and central memory cytotoxic T cells (CD8 T_CM_) (Fig. 3b and Supplementary Table 3e). CD27^+^IgM^−^IgD^−^ switched memory B cells were at the lower limit or reduced (Supplementary Tables 2c and 3c). IgG_1_ and/or IgG_4_ were reduced in P1.2 or P1.1, respectively, T-cell dependent-specific antibodies were reduced in all and EBV-seroconversion was incomplete in P1.1, P2.1 and P2.2 (Supplementary Tables 2a,d and 3a,g). P1.1 and P1.2 had no lymphocyte proliferation when stimulated with tetanus and diphtheria toxoids (Supplementary Table 2e) and P2.1 and P.2.1 did not show any BCG vaccination scar despite reported vaccination (Supplementary Table 3g).

To analyse the patients' T-cell response to EBV, we co-cultured peripheral blood T cells from P2.1 and P2.2, and two EBV^+^ healthy donors (HD) as controls, with autologous irradiated EBV^+^ lymphoblastoid cell lines (LCL) over 10 passages. We found that expansion rates of LCL stimulated T-cell lines (TCL) from P2.1 and P2.2 were significantly lower than from HD (Supplementary Fig. 2a). TCL from P2.1 and P2.2 did not secrete IFN-γ on stimulation with autologous LCL but secreted IFN-γ on stimulation with phytohemagglutintin (PHA) (Supplementary Fig. 2b). IFN-γ secretion of P2.2 TCL after stimulation with human leukocyte antigen (HLA) mismatched LCL suggested that recognition was not major histocompatibility complex (MHC)-restricted (Supplementary Fig. 2b). TCL from P2.1 and P2.2 were unable to secrete IL-4, even after stimulation with PHA (Supplementary Fig. 2c).

In summary, immune phenotypic and functional studies were indicative of a T-cell deficiency with particular absence of T_reg_, insufficient gain of T-cell memory and deficient T-cell response to EBV.

### Genetics

We hypothesized that the T-cell deficiency followed an autosomal recessive Mendelian inheritance pattern with full penetrance (Fig. 2a,e). For family 1, we performed SNP-based homozygosity mapping including the father (F1), mother (M1), P1.1, P1.2 and S1.1. We found four homozygous regions each >1 Mbp on chromosomes 2 (133707180-139877948), 16 (65905023-83076742) and 17 (837260-2754856; 77793860-81049726), that segregated with the disease phenotype (Fig. 2b). Next, we conducted whole-exome sequencing (WES) and combined data sets yielding five candidate variants inside and one outside (*PLCG1*) the homozygous regions (Supplementary Table 4). PolyPhen and SIFT algorithms predicted two variants to be damaging (*WWOX* and *DHODH*) and two to be tolerated (*ZFHX3* and *HSD17B2*). The fifth variant was a gain-of-stop indel mutation in *CARMIL2* and was not analysable by PolyPhen and SIFT algorithms (Supplementary Table 4)^16^,^17^. Since mutations in *WWOX* and *DHODH* cause complex malformations clearly distinct from the patients' phenotype, we focused on *CARMIL2* as the disease causing gene^18^,^19^. We confirmed the autosomal recessive *CARMIL2* c.489insG nonsense variant (p.E163fs*4) by Sanger sequencing and found segregation with the disease phenotype (Fig. 2a,c,h). CARMIL2 immunoblotting in P1.1 and P.1.2 LCL showed absent protein expression (Fig. 2d).

For family 2, we analysed the autosomal recessive *CARMIL2* c.871+1G>T splice-site variant that had been reported as a WES finding^20^. We confirmed *CARMIL2* c.871+1G>T by Sanger sequencing and found segregation with the disease phenotype (Fig. 2e,f). *In silico* analysis predicted the splice-site variant to cause skipping of exon 11 resulting in a frame shift and a premature stop codon (p.D260fs*70) (Fig. 2h). Sanger sequencing of P2.1 *CARMIL2* cDNA detected a splice product lacking exon 11 and CARMIL2 immunoblotting in P2.1 and P2.2 LCL showed absent protein expression (Supplementary Fig. 3 and Fig. 2g).

Thus, in both families homozygous loss-of-protein-expression *CARMIL2* mutations segregated with the disease phenotype.

### CD28-dependent T-cell function and NK and T-cell cytotoxicity

**Figure 4:**
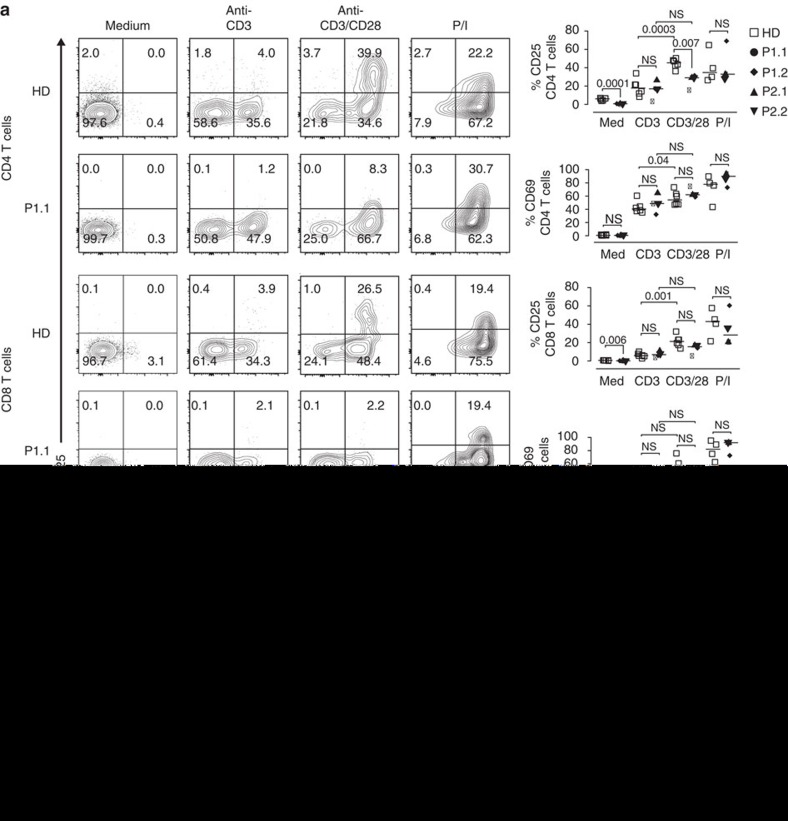
CARMIL2-deficiency impairs CD28-mediated T-cell activation. (**a**) Representative contour plots of CD25 and CD69 surface expression on CD4 and CD8 T cells without (medium) and after stimulation for 48 h with anti-CD3, anti-CD3/CD28 or PMA/ionomycin (P/I). Summary of CD4 and CD8 T-cell CD25 and CD69 surface expression for six HD (open squares), P1.1 (black circle), P1.2 (black rhomb), P2.1 (black up-pointing triangle) and P2.2 (black down-pointing triangle). (**b**) Contour plots of CD25 surface and FOXP3 expression on CD4 T cells without (medium) and after stimulation for 48 h with anti-CD3, anti-CD3/CD28 or PMA/ionomycin (P/I). Summary of CD4 T-cell CD25 surface and FOXP3 expression for four HD and three patients. Corresponding percentages are indicated in each square. Data are representative for four independent experiments with *n*=2 (**a**) or two independent experiments with *n*=2 (**b**). Small horizontal lines indicate the median. Each symbol represents an individual donor. Significance levels are calculated with Welch's *t*-test and indicated in the summary graphs (NS=non significant).

Since murine Rltpr is essential for CD28 co-signalling, we first determined CD28 expression in CARMIL2-deficient patient CD4 and CD8 T cells and found normal surface levels (Supplementary Fig. 4a)^10^. To assess CD28-dependent T-cell activation, we stimulated CARMIL2-deficient PBMC with anti-CD3, anti-CD3/CD28 or PMA/ionomycin (P/I) and measured surface expression of the early activation antigen CD69, the high-affinity interleukin-2 receptor alpha chain CD25 and the T_reg_ master transcription factor FOXP3. Patient CD4 and CD8 T cells showed normal TCR-dependent induction of CD69 and increased CD69 expression after CD28 co-stimulation (Fig. 4a). In contrast, patient CD4 and CD8 T cells lacked upregulation of CD25 in response to anti-CD3/CD28 (Fig. 4a). Furthermore CD25 and FOXP3 co-expression was absent in resting patient CD4 T cells and could not be induced after anti-CD3 and anti-CD3/CD28 stimulation (Fig. 4b).

**Figure 5:**
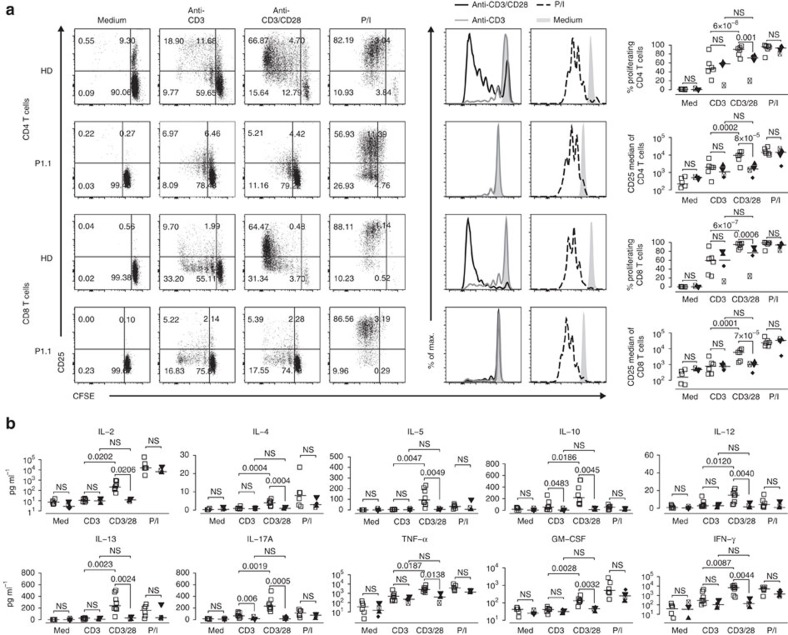
CARMIL2-deficiency impairs CD28-mediated T-cell function. (**a**) Dot and histogram plots of CD25 surface expression and/or CFSE-dilution on CD4 and CD8 T cells without (medium) and after stimulation for 5 days with anti-CD3, anti-CD3/CD28 or PMA/ionomycin (P/I) and summary of CD4 and CD8 T-cell median CD25 MFI and proliferation percentages for six HD and four patients. (**b**) Multiplex cytokine assay for IL-2, IL-4, IL-5, IL-10, IL-12, IL-13, IL-17A, GM-CSF, IFN-γ and TNF-α (pg ml^−1^) in the supernatant collected after 48 h from T cells analysed in **a**. Small horizontal lines indicate the median. Each symbol represents an individual donor. Data are representative of four independent experiments with *n*=4 and pooled supernatants (**b**). Significance levels are calculated with Welch's *t*-test and indicated in the summary graphs (NS=non significant).

To assess CD28-dependent T-cell proliferation and activation-induced cell death (AICD), we applied the same stimulation conditions and analysed CFSE-dilution in combination with CD25 surface expression and 7-AAD cell viability staining. In HD and patient CD4 and CD8 T cells, proliferation was modestly induced after anti-CD3, but only HD T cells showed a robust proliferative response and concomitant CD25 surface expression after CD28 co-stimulation (Fig. 5a). After anti-CD3/CD28 stimulation a small fraction of HD CD4 and CD8 T cells underwent AICD, whereas patient cells did not (Supplementary Fig. 5). In all experiments, HD and patient T cells responded equally towards P/I that are bypassing proximal TCR and CD28 co-signalling.

To assess T-cell effector function, we measured cytokine secretion (IL-2, IL-4, IL-5, IL-10, IL-12, IL-13, IL-17A, GM-CSF, IFN-γ, TNF-α) of stimulated PBMC. On stimulation with anti-CD3, we observed an increase of cytokine concentrations with the exception of IL-10 and IL-17A in patients' PBMC. However, when stimulated with anti-CD3/CD28, a marked increase in cytokine concentrations was only seen in HD PBMC. Stimulation with P/I induced comparable cytokine amounts in HD and patient cell culture supernatants (Fig. 5b).

**Figure 6:**
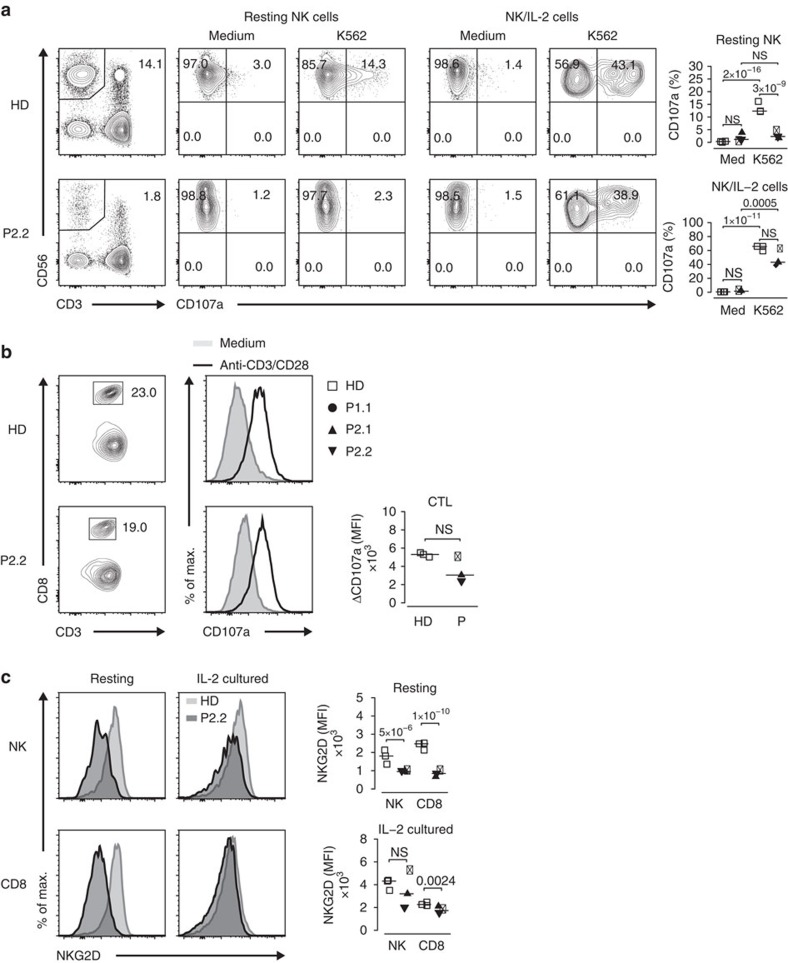
IL-2 rescues degranulation and NKG2D expression on NK and CD8 T cells. (**a**) Contour plots of CD107a expression on resting and IL-2 cultured NK cells without (medium) and after K562 stimulation (K562) and summary of percentages of CD107a expression for three HD (open squares), P1.1 (black circle), P2.1 (black up-pointing triangle) and P2.2 (black down-pointing triangle). (**b**) Contour and histogram plots of CD107a expression on IL-2/phytohemagglutinin cultured CD8 T cells without (medium) and after anti-CD3/CD28 stimulation and summary of percentages of CD107a expression for three HD and three patients. (**c**) Histogram plots of NKG2D expression on resting and IL-2 cultured NK and CD8 T cells and summary of median NKG2D MFI for three HD and three patients. Small horizontal lines indicate the median. Each symbol represents an individual donor. Data are representative of two independent experiments with *n*=3. Significance levels are calculated with Welch's *t*-test and indicated in the summary graphs (NS=non significant).

NK and T-cell cytotoxicity play crucial roles in tumour surveillance and control of EBV^21^,^22^. We therefore determined degranulation of and NKG2D expression on NK and CD8 T cells. NK-cell degranulation in response to co-incubation with the erythroleukemic cell line K562 was impaired and accompanied by a decreased expression of NKG2D. In resting CD8 T cells, NKG2D expression was reduced as well. NK cell and CD8 T-cell pre-culturing with IL-2 abrogated the observed differences in degranulation and NKG2D expression (Fig. 6).

In summary, CARMIL2-deficient CD4 and CD8 T cells show impaired activation, proliferation and effector function in response to CD28 co-stimulation. Degranulation and NKG2D expression were impaired in steady state NK and CD8 T cells, but could be rescued with IL-2.

### CD28 co-signalling

**Figure 7:**
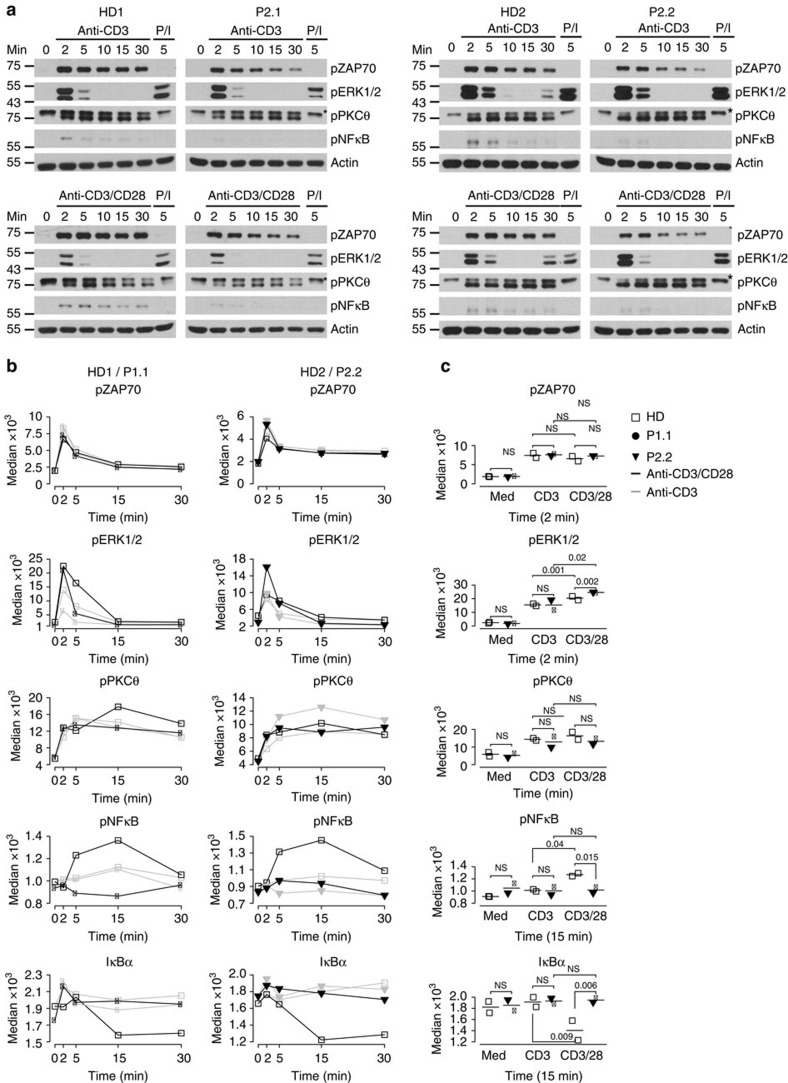
CARMIL2-deficiency impairs CD28 co-signalling. (**a**) Representative immunoblots for pZAP70, pERK1/2, pPKCθ and pNF-κB (p65) with total protein obtained from HD1, HD2, P2.1 and P2.2 T lymphoblasts stimulated with anti-CD3, anti-CD3/CD28 and P/I for 0, 2, 5, 10, 15 and 30 and 5 min, respectively. Actin serves as loading control, molecular weight markers are indicated in kDa and asterisks mark non-specific bands. Data are representative of two independent experiments. (**b**) Median of pZAP70, pERK1/2, pPKCθ, pNF-κB (p65) and IkBa in CD4 T cells of T lymphoblasts from HD1 and HD2 (open squares), P1.1 (black circle) and P2.2 (black down-pointing triangle) stimulated with anti-CD3 and anti-CD3/CD28 for 0, 2, 5, 15 and 30 min by flow cytometry. (**c**) Summary of median (phospho-) protein levels for two HD and two patients at the indicated time points with *n*=3. Significance levels are calculated with Welch's *t*-test (NS=non significant).

The TCR and CD28 co-receptor target an overlapping but not identical array of signalling intermediates leading to joint functional outcomes such as T-cell activation, proliferation and effector function^8^. The TCR recognizes peptide antigens presented by MHC molecules, thus conferring antigen specificity. CD28 interacts with the invariant co-stimulators CD80 and CD86, thus leading to full naive T-cell activation^23^. The precise positioning of CARMIL2 and its interaction partners in CD28 co-signalling are currently unknown^10^. To analyse CD28 co-signalling, we stimulated T lymphoblasts of HD1, HD2, P1.1, P2.1 and P2.2 with anti-CD3, anti-CD3/CD28 for 2, 5, 10, 15 and 30 min and performed immunoblotting and (phospho-) flow cytometry for essential signalling intermediates (Fig. 7 and Supplementary Fig. 6). P/I stimulation for 5 min served as a positive control. Phosphorylation of the upstream tyrosine kinase ZAP70 as well as the serine/threonine kinase PKCθ that participate in the immunological synapse was comparable in HD and patient cells (Fig. 7)^23^. Similarly, there was no difference in phosphorylation of the downstream mitogen-activated protein kinase ERK1/2 in CARMIL2-deficient and HD T lymphoblasts (Fig. 7). In contrast, in CARMIL2-deficient T lymphoblasts, the downstream canonical NF-κB pathway was not activated in response to CD28 co-signalling as illustrated by aberrant degradation of IκBα and phosphorylation of NF-κB p65 (Fig. 7). Signalling data obtained by (phospho-) flow cytometry showed no difference between CD4 and CD8 HD and patient T cells (Fig. 7 and Supplementary Fig. 6).

We conclude that CARMIL2-deficiency selectively impaired the activation of the canonical NF-κB pathway in a CD28-dependent manner.

### Cytoskeleton dynamics and T-cell migration

**Figure 8:**
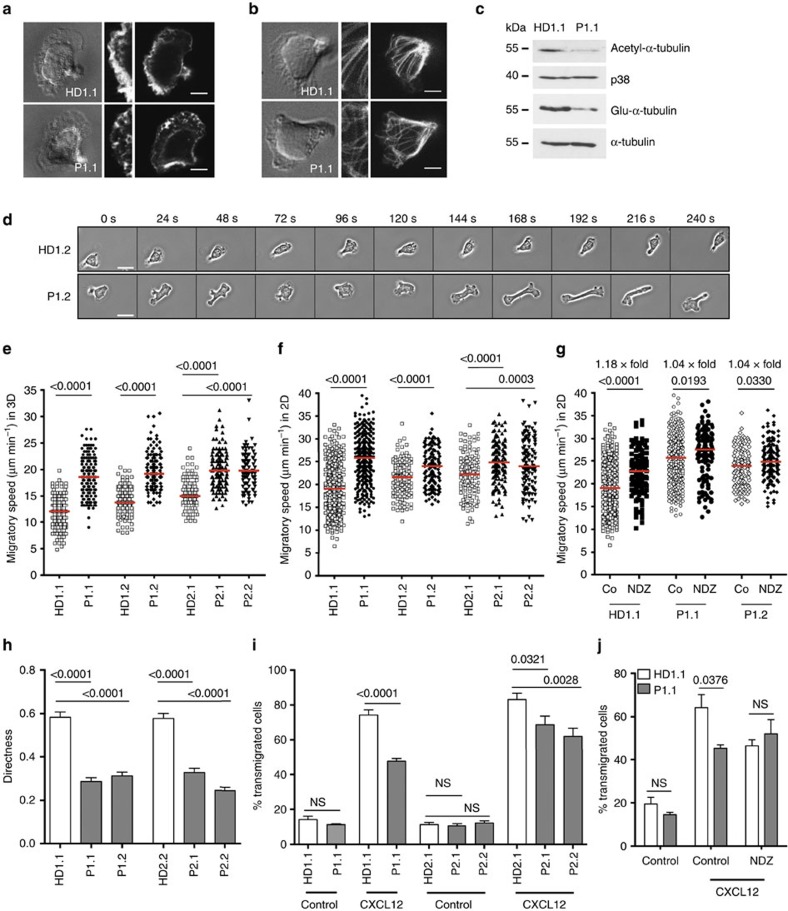
CARMIL2-deficiency leads to defective cytoskeletal organization and migration. Differential interference contrast (DIC) images and fluorescent pictures of F-actin (**a**) and tubulin (**b**) in T lymphoblasts of HD1.1 and P1.1 migrating on ICAM1 (Scale bars, 5 μm). Fluorescent images show the level of the adhesion plane and details thereof are depicted in the middle. (**c**) Representative immunoblots showing stable α-tubulin as detected by acetyl- or detyrosinated glutamyl-α-tubulin in HD1.1 and P1.1. p38 and global α-tubulin serve as loading controls (*n*≥3). (**d**) Time-lapse video microscopy images of HD1.2 and P1.2 T lymphoblasts migrating on ICAM1. Time in seconds is depicted for each frame (Scale bars, 10 μm). (**e**) Spontaneous migration of T lymphoblasts (*n*≥110) in a 3D collagen matrix. For HD1.1, HD1.2, P1.1, P1.2 three and for HD2.1, HD2.2, P2.1, P2.2 two independent experiments are shown. (**f**) Spontaneous migration of T lymphoblasts on ICAM1 in 2D (*n*≥123 in three independent experiments). (**g**) *x*-fold migratory speed of control (co) versus nocodazol (NDZ) pretreated T lymphoblasts is depicted (*n*≥128 in two independent experiments). (**e**–**g**) Red horizontal lines indicate the median. (**h**) Directness of migration on ICAM1 was quantified from T lymphoblast tracks (mean±s.e.m., *n*=99). (**i**,**j**) Chemotactic T-cell migration through 5 μm pores towards CXCL12 was analysed. When indicated T lymphoblasts were pretreated with nocodazole (NDZ). Three (**i**) or two (**j**) independent experiments with two technical replicates were done (mean±s.e.m.). Significance levels are calculated for patient T lymphoblasts in relation to HD within corresponding assays using the two-sided unpaired Student's *t*-test and are indicated in the summary graphs (NS=non significant).

The cytoskeleton plays an important role at almost all stages of the immune response. Not surprisingly, there is a growing list of PID associated with mostly actin-related cytoskeletal defects^24^. Through its CBR domain, CARMIL2 is supposed to play an important role in actin dynamics^25^. To determine the role of CARMIL2 in cytoskeleton dynamics and migration, we analysed total amounts of filamentous (F)-actin and actin distribution in the leading edge of HD and patient T lymphoblasts migrating on ICAM1 in 2D. Quantification of total F-actin was comparable between HD and patient T lymphoblasts in steady state, after PMA-induced polymerization and latrunculin B-induced depolymerization (Supplementary Fig. 7a,b). Surface expression of total and active LFA1—that interacts with ICAM1—and subcellular LFA1 distribution were comparable in HD and patient T lymphoblasts as well (Supplementary Fig. 7c,d). However, CARMIL2-deficient T lymphoblasts migrating on ICAM1 in 2D, showed a dispersed F-actin distribution at the leading edge as compared with a homogeneous distribution in CARMIL2-proficient cells (Fig. 8a and Supplementary Fig. 7e). In addition to the effect on F-actin distribution, localization analysis of α-tubulin showed a markedly disrupted microtubule network (Fig. 8b, Supplementary Fig. 7f and Supplementary Movies 1–4). Furthermore, we detected a global reduction in post-translationally modified stable acetyl- and stable detyrosinated glutamyl-α-tubulin monomers in CARMIL2-deficient T lymphoblasts (Fig. 8c and Supplementary Fig. 7g). Microtubule dynamics are crucial for migratory cell polarity and microtubule depolymerization is associated with increased spontaneous motility in immune cells^26^. Accordingly, patient T lymphoblasts showed dispersed polarity (Fig. 8d) and increased spontaneous migratory speed both in a 3D collagen gel and in 2D on ICAM1 (Fig. 8e,f). Pharmacological microtubule disruption with nocodazole (NDZ) increased migratory speed of HD cells in 2D but had only mild effects on CARMIL2-deficient cells suggesting that they already displayed an intrinsic microtubule disruption (Fig. 8g). Although migrating with increased speed that resulted in higher accumulated migratory distance (Supplementary Fig. 7h), CARMIL2-deficient T-cell migration was less orientated with a markedly decreased directness (Fig. 8h) and reduced Euclidean distance (Supplementary Fig. 7i,j). Reduced straightness of patient T-cell migration was further shown by impaired CXCR4-mediated and CXCL12-guided chemotaxis (Fig. 8i). The reduced chemotactic response could not be attributed to aberrant expression of cell surface CXCR4 (Supplementary Fig. 7k). Again, NDZ treatment reduced CXCL12-guided chemotaxis in HD but not patient T lymphoblasts (Fig. 8j).

In summary, CARMIL2-deficient T cells showed impaired F-actin distribution at the leading edge and disturbed microtubule network that were associated with increased LFA1-mediated migratory speed in 2D and LFA1-independent migratory speed in a 3D collagen network. Accumulated migratory distance was increased, but Euclidean distance and migration directness as well as chemokine-guided migration were decreased.

## Discussion

We here describe a novel human PID caused by autosomal recessive mutations in *CARMIL2* that abrogated protein expression. In the context of preserved canonical TCR signalling and CD28 surface expression, CARMIL2-deficient T cells showed impaired CD28-mediated co-signalling. Whereas upstream phosphorylation of ZAP70 and PKCθ and downstream phosphorylation of ERK1/2 were inducible in CARMIL2-deficient T cells, the activation of the canonical NF-κB pathway was impaired. Human CARMIL2-deficiency is reminiscent of a murine model of Rltpr-deficiency discovered in a *N*-ethyl-*N*-nitrosourea-mutagenesis screen^10^. Rltpr-deficient mice also display defective CD28-mediated co-signalling, associated with defective microclustering of Pkcθ at the immunological synapse^10^. We could document regular phosphorylation of PKCθ, but were unable to assess microclustering. It is possible that CARMIL2 contributes to the stabilization of activated PKCθ-microclusters at the immunological synapse and thereby couples CD28 co-signalling to the canonical NF-κB pathway^10^,^27^. However, precise mechanistic details on the role of CARMIL2 for CD28-mediated co-signalling and potentially other signalling pathways need to be addressed in future studies.

On the cellular level, CARMIL2-deficiency did not interfere with thymic development of conventional CD4 and CD8 T cells. This is in line with preserved TCR signalling^2^. In contrast, CARMIL2-deficiency is characterized by impaired naive T-cell activation, proliferation, effector function and insufficient gain of T-cell memory, indicative of a globally compromised peripheral T-cell immunity.

Consistent with an established role of murine CD28 co-signalling for the induction of Foxp3 and the development of natural T_reg_, human CARMIL2-deficient T cells, that were able to express the TCR-inducible activation marker CD69, did not express CD25 and FOXP3 in response to CD28 co-signalling^28^. Similar to murine Rltpr-deficiency, human CARMIL2-deficient patients were devoid of T_reg_ in the peripheral blood^10^. At first sight, it may appear surprising, that a lack of T_reg_ in CARMIL2-deficiency was not associated with immune dysregulation. However, as discussed before and as shown by our data, CD28 co-signalling is necessary to fully activate naive T cells and to induce proliferation and effector T-cell (T_eff_) responses^23^. Therefore, the complete absence of T_reg_ could be counterbalanced functionally by impaired T_eff_ responses.

CARMIL2 has been shown to provide a functional link between vimentin intermediate filaments and membrane-associated actin networks during lamellipodia formation, cell migration and invadopodia-mediated matrix degradation in fibrosarcoma cells^29^. Our studies in CARMIL2-deficient T lymphoblasts showed impaired actin distribution in the leading edge and a severely disturbed microtubule network associated with increased spontaneous migratory speed but decreased directness and chemokine-directed migration. It is possible that high migratory speed of CARMIL2-deficient T cells results in suboptimal scanning of peptide:MHC-complexes on professional antigen-presenting cells and thus affects antigen-specific T-cell activation^30^. In line with that, we found impaired proliferation and cytokine production in co-cultures of antigen-presenting autologous EBV^+^ LCL and CARMIL2-deficient TCL. However, keeping in mind the impaired canonical NF-κB pathway discussed before, it is difficult to weight the particular contributions of disturbed antigen-scanning and CD28 co-signalling on T-cell activation. Despite increased migratory speed of CARMIL2-deficient T cells, impaired directness and chemokine-mediated migration might additionally impair T_eff_ responses in CARMIL2-deficient patients. Defective microtubule stability has been associated with increased spontaneous migration in 3D networks and decreased chemokine-guided migration of T cells^31^,^32^. Thus, CARMIL2 now emerges as a critical regulator of cytoskeletal dynamics in T cells. At this point, we cannot demarcate specific effects of defective T-cell activation and defective T-cell migration accounting for the state of immunodeficiency.

The most prominent clinical finding in all four CARMIL2-deficient patients was EBV^+^ SMT. EBV^+^ SMT is a very rare tumour entity usually associated with secondary immunodeficiency disorder, for example, in the context of HIV-infection or iatrogenic immunosuppression in organ transplant patients^15^.

In human OX40-deficiency, HHV8^+^ Kaposi's sarcoma cells strongly expressed OX40L and impaired OX40 signalling on immune cells was supposed to be a key pathogenic factor^3^. However, we did not detect increased expression of CD80 or CD86 on EBV^+^ SMT.

In human SAP, CD27 and CD70 co-signalling deficiencies, orthotopic EBV-infection causes hemophagocytic lymphohistiocytosis (HLH) and/or highly proliferating and immunogenic B-cell lymphomas, respectively^4^. In striking contrast, CARMIL2-deficiency is not associated with EBV-induced B-cell proliferation, but rather with a slowly proliferating and pauci-immune SMT associated with dystopic EBV-infection^33^. Since CD28 co-signalling is essential to activate naive T cells and to induce T_eff_ responses, EBV^+^ SMT in the context of CARMIL2-deficiency might evade anti-tumour immunity and indicate a particular importance of CD28 co-signalling in controlling this tumour entity^23^. On the other side, even in the absence of EBV^+^ lymphoproliferative disorder, we found that CARMIL2-deficient T cells also had decreased capacity to recognize EBV^+^ autologous LCL. Further studies may unravel why EBV causes leiomyomatosis rather than B-cell proliferation in CARMIL2-deficient patients.

Of note, exposure to recombinant IL-2 partially restored markers of NK and cytotoxic T-cell function that are crucial to prevent EBV-triggered lymphoproliferative disorders^34^. This observation might not only be relevant for potential immunomodulatory effects *in vivo*, it further might indicate that besides CD28 co-signalling additional receptor systems could depend on CARMIL2. From a technical point of view, IL-2 responsiveness also severely hampered our attempts to reconstitute the T-cell phenotype *in vitro* by lentiviral gene transfer.

In summary, CARMIL2-deficiency is a novel primary autosomal recessive human immunodeficiency disorder highlighting a role for CARMIL2 as orchestrator of CD28 co-signalling and cytoskeletal dynamics necessary for T_reg_ development, adequate T-cell activation, proliferation, differentiation, effector function as well as T-cell polarity and migration.

## Methods

### Patients

Patients were referred to the Dr von Hauner Children's Hospital, Ludwig-Maximilians-Universität, Munich, Germany and the Department of Pediatric Gastroenterology, Federal University of Minas Gerais, Belo Horizonte, Brazil. Informed consent was obtained according to current ethical and legal guidelines. The study protocol was approved by the Institutional Review Board at the University Hospital, Ludwig-Maximilians-Universität, Munich, Germany (Project Number 381-11). The study was conducted in accordance with the Declaration of Helsinki.

### LCL-responsive T-cell lines

For initiating T-cell lines (passage 1), 1 × 10^6^ peripheral blood mononuclear cells (PBMC) from patients P2.1 and P2.2 and from two EBV-seropositive healthy donors (HD1 and HD2) were co-cultured with gamma-irradiated (80 Gy) autologous EBV^+^ lymphoblastoid cell lines (LCL) at a 10:1 ratio (PBMC:LCL) in a total volume of 2 ml T-cell medium consisting of AIM-V lymphocyte medium supplemented with 10% pooled human serum, 2 mM L-glutamine, 10 mM HEPES and 1% amphotericin B. To prevent expansion of FCS-reactive T cells, stimulator LCL were continuously cultured in RPMI 1640 medium supplemented with 10% human serum, 2 mM L-glutamine, 1% nonessential amino acids, 1 mM sodium pyruvate, 2 mM L-glutamine and 50 mg ml^−1^ gentamicin as described^35^. After 48 h, 50 U ml^−1^ IL-2 (Novartis) was added to the culture medium. The T-cell lines were restimulated every two weeks in the same way and split when needed. After ten passages, the lines contained predominately (>95%) CD3^+^CD8^+^ cells and few (<5%) CD3^+^CD4^+^ cells. Specificity of T cells was tested by incubating 5 × 10^4^ target cells (autologous or allogeneic HLA-mismatched LCL) with 5 × 10^4^ T cells in a final volume of 200 μl T-cell medium. After 24 h of co-culture, ELISA (R&D Systems) for measuring supernatant cytokines was performed.

### Genetic analysis

Genomic DNA of family 1 subjects F1, M1, P1.1, P1.2 and S1.1 (Fig. 2a) was genotyped using the Genome-wide Human SNP array 6.0 on a GEO Platform GPL6801 (Affymetrix) and used to prepare exome libraries using the SureSelect XT Human All Exon V4+UTRs kit (Agilent Technologies). Barcoded libraries were sequenced on a SOLiD 5500 XL next-generation sequencing platform (Life Technologies) at the Dr von Hauner Children's Hospital sequencing facility to an average coverage depth of 80 reads. Bioinformatic data processing followed an in house pipeline: Reads were aligned to the human reference genome (version hg19) using LifeScope Analysis Suite 2.5.1 (Life Technologies, Carlsbad, CA, USA) using standard parameters. The lane data for each sample were merged and the PCR duplicates were removed using SAMtools version 1.18 (ref. 36). SAMtools was also used to call SNPs with the command: ‘mpileup –M 300 –I –g –D -C50 –S'. Small insertions and deletions (indels) were identified using DinDel version 1.01 (ref. 37), by first identifying putative indels for each sample Individually, and then re-running the DinDel on the pooled set of samples from family A with the previously identified indels as suggested variants. Variants were annotated and their manifestation at the mRNA and protein level identified according to the Ensembl Database (version 68). In addition, information on variant frequencies was collected from HapMap (HM)^38^, 1000 genomes (TG)^39^, NHLBI Grand Opportunity Exome Sequencing Project (NH) and ExAC^40^.The WES approach for genomic DNA of family 2 subjects F2, M2, P2.1 and P2.2 has been published before^20^. The c.489insG and c.871+1G>T *CARMIL2* mutations were confirmed by Sanger sequencing (see Supplemantary Table 5 for primer information). *CARMIL2* cDNA was reverse transcribed from P2.1 T lymphoblast total mRNA (RevertAid H Minus Reverse trascriptase; ThermoFisher Scientific) and skipping of exon 11 was confirmed by Sanger sequencing (see Supplemantary Table 5 for primer information).

### Cell culture and stimulation

PBMC were isolated from healthy donors and patients by Ficoll-Hypaque (Pharmacia) density gradient centrifugation. PBMC were maintained in RPMI 1640 supplemented with 2 mM glutamine, 100 U ml^−1^ penicillin, 100 μg ml^−1^ streptomycin and 10% FCS (ThermoFisher) in a 5% CO_2_ incubator at 37 °C. Cell lines have been tested negative for mycoplasma by PCR using VenorGeM Mycoplasma Detection Kit (MP0025, Sigma-Aldrich). Stimulation titration curves were established (Supplementary Fig. 4b). For T-cell activation, proliferation and cytokine secretion experiments PBMC were stimulated with anti-CD3-coupled beads (Bio-anti-CD3, OKT3 from eBioscience coupled with anti-Biotin MACSiBeads from Miltenyi Biotec) at a ratio of 10:1 with and without 1 μg ml^−1^ soluble anti-CD28 (CD28.2, eBioscience) or with 0.5 ng ml^−1^ phorbol 12-myristate 13-acetate (PMA) and 1 μM ionomycin (Sigma-Aldrich). For T-cell signalling analysis by immunoblotting and (phospho-) flow cytometry, T lymphoblasts were induced from PBMC with 5 ng ml^−1^ PMA, 1 μM ionomycin and 100 U ml^−1^ IL-2 (Novartis) for two days and cultured for 8–16 additional days in complete RPMI 1640 substituted with 100 U ml^−1^ IL-2. For T-cell migration assays and analysis of cytoskeletal proteins, PBMCs were activated for four days with 1 μg ml^−1^ phytohemagglutinin (Remel, Dartford). Cells were then maintained in medium containing 20 ng ml^−1^ recombinant IL-2 (Proleukin, Novartis). Activated human T lymphoblasts were used at day 10–14 following stimulation.

### Flow cytometry

T_reg_ cells were stained with APC-H7-anti-CD3 (SK7, 1:50), APC-anti-CD4 (SK3, 1:100), PE-anti-CD25 (M-A251, 1:50) (all BD Biosciences) and FITC-anti-CD127 (eBioRDR5, 1:50, eBioscience). Intracellular staining with PerCP-Cy5.5-anti-FOXP3 (PCH101, 1:25) was performed using FOXP3 staining kit (eBioscience). Memory T cells were surface stained with APC-H7-anti-CD3 (SK7, 1:50), PerCP-anti-CD4 (SK3, 1:10), PacB-anti-CD8 (RPA-T8, 1:100), APC-anti-CD28 (CD28.2, 1:200), PE-Cy7-anti-CD45R0 (HI100, 1:100), PE-anti-CCR7 (3D12, 1:5) (all BD) and FITC-anti-CD27 (O323, 1:50, eBioscience). Proliferative response and AICD were measured by labelling PBMC with 2.5 μM carboxyfluorescein diacetate succinimidyl ester (CFSE, ThermoFisher), 7-aminoactinomycin D (7-AAD, 2.5 μg ml^−1^, BD), APC-H7-anti-CD3 (SK7, 1:50), PE-anti-CD25 (M-A251, 1:50), APC-anti-CD8 (SK1, 1:200) (all BD) and PC5-anti-CD4 (13B8.2, 1:100, Beckman Coulter) 5 days after stimulation. Degranulation of NK cells and CTLs (cytotoxic T lymphocytes) was determined by surface staining with PerCP-anti-CD3 (SK7, 1:100), FITC-anti-CD8 (SK1, 1:100), APC-anti-CD56 (NCAM16.2, 1:100), PE-anti-CD107a (H4A3, 1:50) and PE-Cy7-anti-NKG2D (1D11, 1:20) (all BD). Expression analysis of total and high-affinity LFA1 was performed with conformation-specific anti-LFA1 monoclonal antibodies clone 38 (AbD Serotec) and clone 24 (kind gift from N. Hogg) and mouse IgG1 (MOPC-21) from Biozol (Eching) as isotype control at 37 °C, 7% CO_2_, followed by goat anti-mouse IgG-APC (BioLegend) on ice, all used at concentration of 10 μl ml^−1^. CXCR4 was detected with PE-anti-CXCR4 (clone 44717, 10 μl per 10^6^ cells, R&D Systems). For (phospho-) flow analysis, 2.5 × 10^6^ T lymphoblasts were stimulated with titrated antibody concentrations (Supplementary Fig. 6a) by cross-linking of 1 μg ml^−1^ anti-CD3 (UCHT1) and/or 10 μg ml^−1^ anti-CD28 (CD28.2) with 10 μg ml^−1^ goat anti-mouse IgG all from BD for the indicated time. Cells were fixed and permeabilized with fixation and permeabilization kit from Molecular Probes in 90% methanol. Before staining of surface antigens, unbound goat anti-mouse IgG was blocked with normal mouse IgG (Invitrogen). The permeabilized cells were stained with the following titrated intracellular antibodies detecting: ERK1/2 phosphorylated at T202 and Y204 (#4377, 197G2, 1:200), ZAP70 phosphorylated at Y319 (#2717, 65E4, 1:400), PKCθ phosphorylated at T538 (#9377, 1:200), rabbit AlexaFluor488-conjugated secondary antibody (#4412, 1:1000) all purchased from Cell Signalling and AlexaFluorA647-anti-NFκB p65 phosphorylated at S529 (K10-895.12.50, 1:10) and AlexaFluorA647-anti-IκBα (25/IkBa/MAD-3, 1:10) both purchased from BD. Stained cells were analysed using a BD LSRII Fortessa or BD CANTOII flow cytometer. Gating strategies are shown in Supplementary Fig. 11. Data analysis was performed with FlowJo software (TreeStar).

### Cytokine analysis

Culture supernatants were collected 48 h after stimulation, pooled from four technical replicates and assayed for IL-2, IL-4, IL-5, IL-10, IL-12, IL-13, IL-17A, TNF-α, GM-CSF and IFN-γ using a 10-plex cytokine assay kit (L500000CA5) on a Bio-Plex 200 System (BioRad) following the manufacturer's instructions.

### NK and CD8 T-cell degranulation and NKG2D expression

Degranulation of resting and activated NK cells was measured by surface staining of CD107a without (medium-cultured cells) and 3 h after stimulation with K562 cells at a ratio of 1:1 (ref. 41). The erythroleukemic cell line K562 (ATCC, CCL-243) was used as target cell line. NK cells were cultured in medium containing 600 U ml^−1^ IL-2 (Novartis) for 48 h to assess degranulation of activated NK cells. CTL (cytotoxic T lymphoblasts) degranulation was evaluated in T lymphoblasts 48 h after stimulation with 1.25 μg ml^−1^ phytohemagglutinin-L (PHA-L, Sigma) and 200 U ml^−1^ IL-2 (Novartis). CTL degranulation was calculated by the difference in median fluorescence intensity of CD107a of CTLs stimulated with CD3/CD28 coated microbeads (ThermoFisher Scientific) at a ratio of 1:10 for 3 h and medium-cultured cells.

### Immunoblotting and CD28 co-signalling

For CARMIL2 expression, CD28 co-signalling and cytoskeletal analyses cells were left unstimulated or stimulated as mentioned above, respectively. Protein isolation, SDS-PAGE and immunoblotting were performed as published elsewhere^42^. The following primary and secondary antibodies were used: CARMIL2 (HPA041402, 1:400, Sigma-Aldrich and ab122717, 1:400, Abcam), actin (sc-8432, 1:500, Santa Cruz), detyrosinated α-tubulin (Glu-tubulin, AB3201, 1:500, Millipore), acetylated α-tubulin (6-11-B-1, ab24610, 1:1,000, Abcam), α-tubulin (DM1A, CP06,1:2,000, Calbiochem), vinculin (V9131, 1:10,000, Sigma-Aldrich), p38 (#9212; 1:1,000, Cell Signalling) and goat-anti-mouse-HRP (1:4,000) and goat-anti-rabbit-HRP (1:5,000) both purchased from Jackson ImmunoResearch. For (phospho)-protein analysis, the generation and stimulation of T lymphoblasts was carried out as described above and the following antibodies were used: ERK1/2 phosphorylated at T202 and Y204 (#4377, 197G2, 1:1,000), ZAP70 phosphorylated at Y319 (#2717, 65E4, 1:1,000), PKCθ phosphorylated at T538 (#9377, 1:1,000), NF-κB phosphorylated at S536 (#3033, 1:1,000) and rabbit HRP-conjugated secondary antibody (#7074, 1:3,000) all purchased from Cell Signalling. Uncropped immunoblots are shown in Supplementary Figs 8–10.

### Quantification of F-actin

To quantify the amount of F-actin, T lymphoblasts were left untreated or were incubated with 100 nM phorbol-myristate-acetate (PMA, Sigma-Aldrich) for 5 min or with 0.5 μM latrunculin B (Calbiochem) for 10 min at 37 °C followed by fixation with 4% paraformaldehyde. Fixed cells were permeabilized with 0.01% Triton X-100 (Sigma-Aldrich), stained with phalloidin-Alexa594 (1:500, Invitrogen) and F-actin amount was quantified by flow cytometry analysis using a BD FACSCalibur and FlowJo software (Tree Star).

### Migration assays

Two-dimensional migration of T lymphoblasts on ICAM1 were analysed on μ-slides (Ibidi) coated with 0.15 μg recombinant human ICAM1/Fc (R&D Systems) per channel over night at 4 °C. Slides were blocked with 1% BSA/PBS for 1 h before use. A total of 5 × 10^4^ T lymphoblasts per channel were allowed to migrate in a steam-saturated incubation chamber at 37 °C, 7% CO_2_, and images were taken every 8 s for 1 h with a Plan-Apochromat 40 × /1.3 NA oil objective using an Axiovert 200 microscope (all Zeiss Microscopy). The migratory speed, accumulated and Euclidean distance and directness were quantified using ImageJ software (National Institute of Health) and the chemotaxis and migration tool V2.0 from Ibidi. Cells migrating <80 μm in 30 min were not included as they were mostly stationary. To analyse the three-dimensional migration of T lymphoblasts, a collagen matrix was prepared using 1 mg ml^−1^ collagen type I from rat tail according to the manufacturer's instructions (Ibidi). Activated T lymphoblasts were embedded into the collagen matrix at a concentration of 1 × 10^5^ cells within a μ-slide. Analysis and quantification of cell migration was performed as described above by taking pictures every 8 s for 30 min using an EC Plan-Neofluar 20 × /0.50 NA. Migration assays in response to CXCL12 were performed using 6.5 mm transwells with 5 μm pore size (Corning). 5 × 10^5^ T lymphoblasts were added on the top of each filter. Medium containing 2.5% serum and 25 mM Hepes was placed at the bottom and supplemented with 100 ng ml^−1^ CXCL12 (R&D Systems) when indicated. Assays were run for 2 h at 37 °C and 7% CO_2_. Migrated lymphocytes were collected and quantified using CountBright Absolute Counting Beads (Molecular Probes) and a BD FACSCalibur.

### Immunofluorescence imaging

Immunofluorescence staining of migrated cells was performed on 15 mm glass coverslips, coated with 0.7 μg recombinant human ICAM1/Fc (R&D Systems). Migration of 1 × 10^5^ T lymphoblasts was allowed for 1 h at 37 °C, 7% CO_2_ in HBSS/25 mM HEPES. Adherent cells were fixed with 3% paraformaldehyde, permeabilized with 0.1% Triton X-100 and coverslips were incubated with an anti-LFA1 antibody (clone 38, 10 μg ml^−1^, AbD Serotec) followed by goat-anti-mouse-Alexa 488 (Molecular Probes; 1:150) or Phalloidin-Alexa 546 (1:100, Invitrogen). For localization of tubulin, polarized T cells were fixed in half-concentrated PHEM buffer (30 mM PIPES, 12.5 mM HEPES, 5 mM EGTA, 1 mM MgCl_2_; pH 6.9) freshly supplemented with 0.25% glutaraldehyde and 0.25% Triton X-100 for 5 min. Unreacted glutaraldehyde was quenched for 10 min with 1 mg ml^−1^ of freshly dissolved sodium borohydride and T cells were subjected to anti-tubulin antibody (YL12, ab6160, 1:200 Abcam), followed by goat-anti-rat-Cy3 (1:200, Jackson ImmunoResearch). ProLong Gold Antifade Reagent (Life Technologies) was used as mounting medium. Fixed samples were imaged at room temperature with an ApoTome-attached Observer Z1, and a Plan-Apochromat 100 × /1.4 NA oil objective (Zeiss Microscopy). Bright-field images were performed using differential interference contrast (DIC). An AxioCam MRm camera and the AxioVision Software (Zeiss Microscopy) were used for image acquisition and image analysis.

### Immunohistochemistry and *in situ* hybridization

For immunohistochemistry, the following antibodies and dilutions were used on a Ventana Benchmark XT autostainer with ultraView Universal DAB detection kits (Ventana Medical Systems): muscle specific actin (DAKO; HHF35; 1:200), calponin (DAKO; CALP; 1:300), pan-cytokeratin (Beckman Coulter; KL-10; 1:150), desmin (DAKO; D33; 1:300), CD34 (Cell Marque; QBEnd/10; 1:800), CD117 (DAKO; A4502; 1:400), S100 (DAKO; Z0311; 1:3,000), Ki67 (DAKO; MIB-1; 1:150), CD80 (Abcam; EPR1157(2); 1:300), p53 (Leica Microsystems; DO-7; 1:25), C-MYC (Abcam; Y69; 1:100), LMP1 (Diagnostic BioSystems; CS1/CS2/CS3/CS4; 1:100), LMP2A (Acris antibodies; 15F9; 1:100), EBNA2 (Merck Millipore; R3; 1:25) and the viral transcription factor ZEBRA (Santa Cruz Biotechnology; BZ1; 1:100). CD86 (BIOZOL Diagnostica; 1B3; 1:100) was stained manually. Chromogenic *in situ* hybridization for EBV-encoded RNA (EBER) was performed using fluorescein-labeled oligonucleotide probes (INFORM EBER Probe, Ventana). Fluorescence *in situ* hybridization for C-MYC (Zyto Vision) was performed as described before^43^.

### Statistical analysis

Data were analysed with the software environment R (version 3.2.3) or Prism 5 (GraphPad) for statistical computing and graphics. Normal Gaussian distribution of data sets was tested using Shapiro-Wilk normality test. Significances between two groups were calculated with the two-sided unequal variances Welch's *t*-test or two-sided unpaired Student's *t*-test. Two-way ANOVA was used for comparisons of more than two groups. *P* values of <0.05 were considered significant.

### Data availability

CARMIL2 variant data that support the findings of this study have been deposited in ClinVar with the primary accession codes SCV000322745 and SCV000322755. Other data that support the findings of this study are available from the corresponding author upon request.

## Additional information

**How to cite this article**: Schober, T. *et al*. A human immunodeficiency syndrome caused by mutations in *CARMIL2*. *Nat. Commun.* **8**, 14209 doi: 10.1038/ncomms14209 (2017).

**Publisher's note**: Springer Nature remains neutral with regard to jurisdictional claims in published maps and institutional affiliations.

## Supplementary Material

Supplementary Information Supplementary Figures and Supplementary Tables

Supplementary Movie 1 Localization of tubulin in polarized T cells from HD2.1.

Supplementary Movie 2 Localization of tubulin in polarized T cells from HD2.2.

Supplementary Movie 3 Mislocalization of tubulin in polarized CARMIL2-deficient T cells from P2.

Supplementary Movie 4 Mislocalization of tubulin in polarized CARMIL2 deficient T cells from P2.2.

## Figures and Tables

**Table 1:** Clinical features of CARMIL2-deficient patients.

	**P1.1**	**P1.2**	**P2.1**	**P2.2**
Origin	Yemen	Yemen	Brazil	Brazil
Consanguinity	Yes	Yes	Yes	Yes
*CARMIL2* mutation	c.489insG	c.489insG	c.871+1G>T	c.871+1G>T
CARMIL2 protein expression	No	No	No	No
Failure to thrive	Yes*	Yes†	Yes‡	Yes§
EBV^+^ SMT	Gut	Gut, liver, spleen, kidney, brain	Gut, liver, lung	Gut, liver
Age at diagnosis of EBV^+^ SMT	7 years	4 years	9 years	6 years
Infections	Recurrent upper airway infections	Recurrent upper airway infections	Recurrent *Giardia* spp., recurrent (retention)-pneumonia	Recurrent chickenpox, pneumonia
Chronic diarrhoea	No	Yes	Yes	No
Skin symptoms	Eczematous dermatitis, skin warts	Recurrent abscesses with *S. aureus*	Skin warts	Eczematous dermatitis
Current state	Progressive disease	Deceased	Deceased	Deceased

^*^Age 7y8m: weight 17.7 kg, *Z* score −2.2; height 115 cm, *Z* score −1.8; head circumference 51 cm, *Z* score −0.5.

^†^Age 5y2m: weight 7.4 kg, *Z* score −5.5; height 85 cm, *Z* score −4.4; head circumference 45 cm, *Z* score −3.8.

^‡^Age 8y0m: weight 14 kg, *Z* score −6.2; height 105 cm, *Z* score −4.4; head circumference not determined.

^§^Age 12y2m: weight 14.5 kg, *Z* score −7.23; height 116 cm, *Z* score −4.59; head circumference 50 cm, *Z* score −3.26.
