# Supplementary material for: A human immunodeficiency syndrome caused by mutations in CARMIL2
Source: Nat Commun. 2017 Jan 23;8:14209. doi: 10.1038/ncomms14209 (PMC5473639; doi:10.1038/ncomms14209)
Supplement: Supplementary Information — Supplementary Figures and Supplementary Tables [file ncomms14209-s1.pdf]

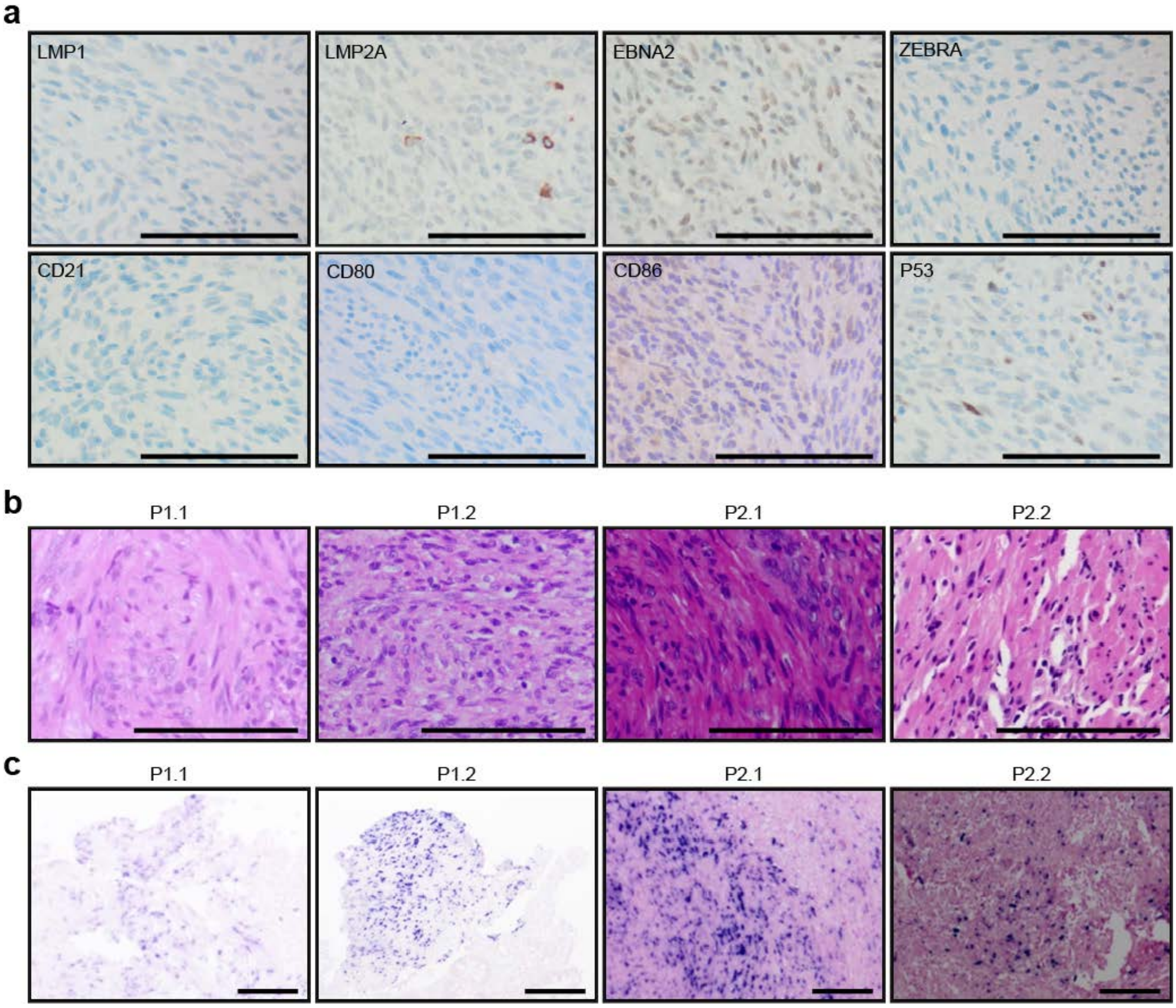


**Supplementary Figure 1.** EBV$^+$ SMT show latency type III. (**a**) P2.1 immunohistochemistry of the EBV proteins LMP1 (negative), LMP2A (scattered positive), EBNA2 (partially positive), ZEBRA (negative), of the EBV B cell entry receptor CD21 (negative), the T cell co-stimulators CD80 and CD86 (negative) and the tumor suppressor P53 (not overexpressed). (**b**) P1.1, P1.2, P2.1 and P2.2 hematoxylin and eosin (H&E) stains with leiomyogenic tumor cells and (**c**) EBER *in situ* hybridization. Scale bars indicate 100µm each.

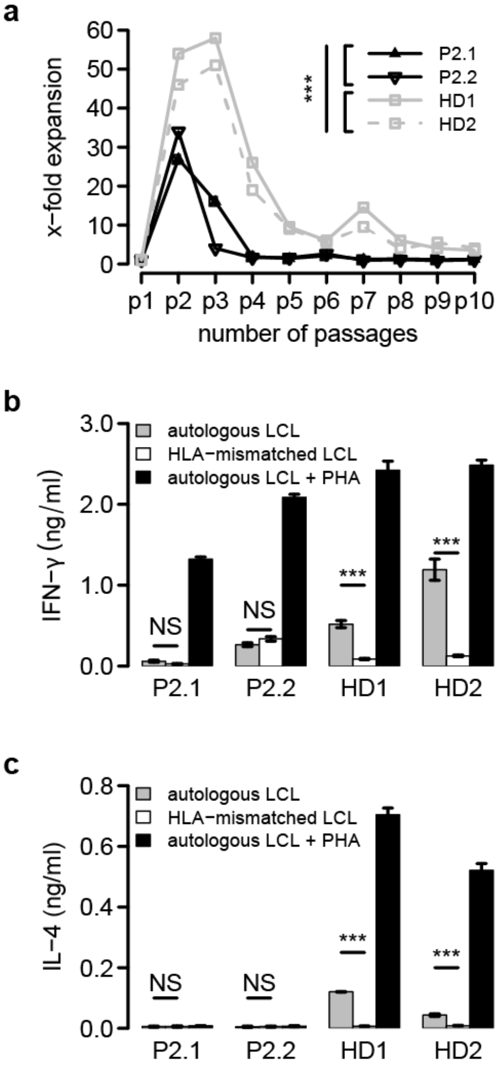


**Supplementary Figure 2.** CARMIL2-deficient TCL do not respond to autologous $EBV^+$ LCL. T cell lines (TCL) from $EBV^+$ HD1, HD2, P2.1 and P2.2 were repeatedly stimulated with autologous $EBV^+$ lymphoblastoid cell lines (LCL). (**a**) Growth kinetics of TCL over ten passages (p). (**b**) IFN-γ and (**c**) IL-4 secretion of TCL after ten passages upon co-culture with autologous or HLA-mismatched $EBV^+$ LCL, or after antigen-independent activation with phytohemagglutinin (PHA). Significance was determined by using two-way ANOVA (**a**), or the two-tailed unpaired *t*-test analysis (**b**) and (**c**). Error bars, mean ± s.e.m. with n=3. NS = non significant. *** $p < 0.0001$.

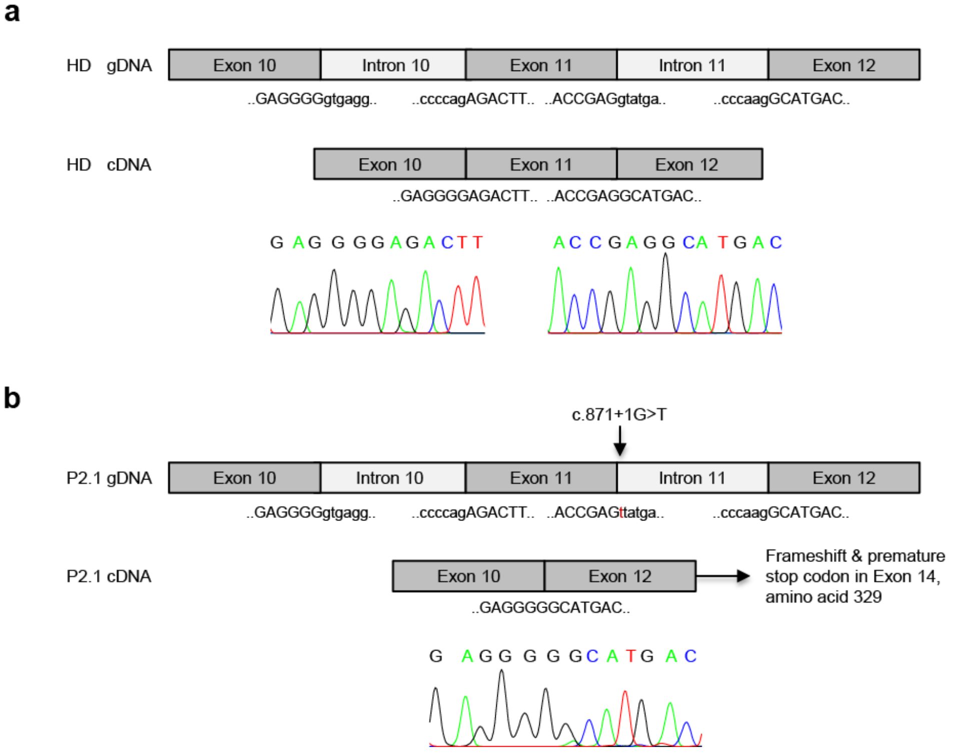


**Supplementary Figure 3.** *CARMIL2* c.871+1G>T leads to skipping of exon 11 and a premature stop codon. (**a**) Schemes of wildtype (HD) *CARMIL2* gDNA with exon-intron boundaries and cDNA with exon-exon boundaries and corresponding cDNA electropherogram. (**b**) Schemes of mutant c.871+1G>T (P2.1) *CARMIL2* gDNA with exon-intron boundaries and cDNA with exon-exon boundaries and corresponding cDNA electropherogram. The G>T transition is depicted in red, exonic bases in capital and intronic bases in small letters.

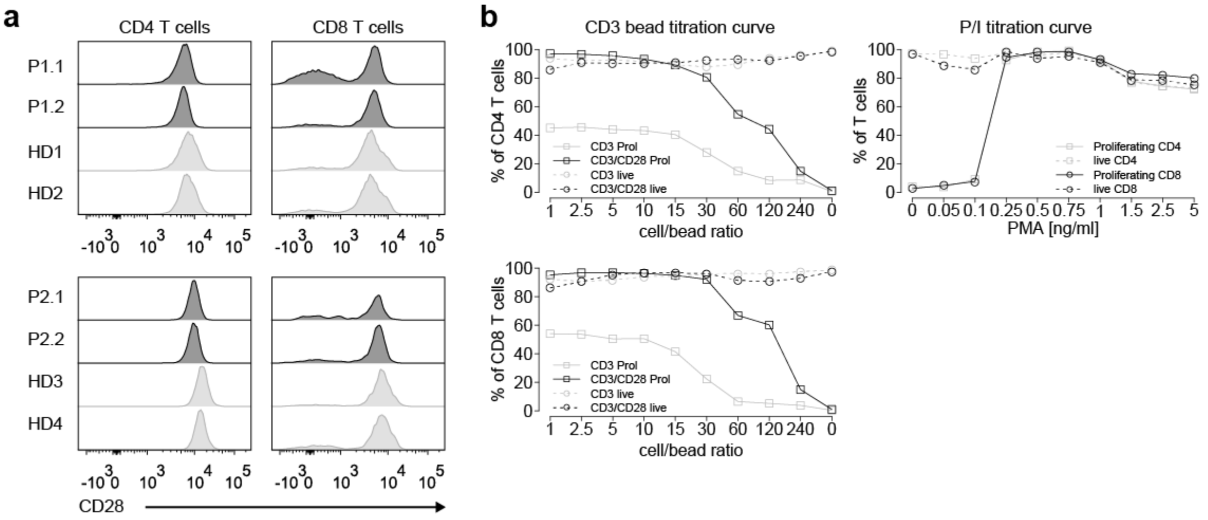


**Supplementary Figure 4.** CARMIL2-deficiency does not interfere with CD28 expression. (**a**) Representative histogram plot overlay of CD28 surface expression for four HD and four patients. (**b**) CFSE dilution and live cell rate determined by 7-AAD exclusion in CD4 and CD8 T cells stimulated for five days with decreasing anti-CD3 bead to cell ratios and PMA/ionomycin with increasing PMA concentrations.

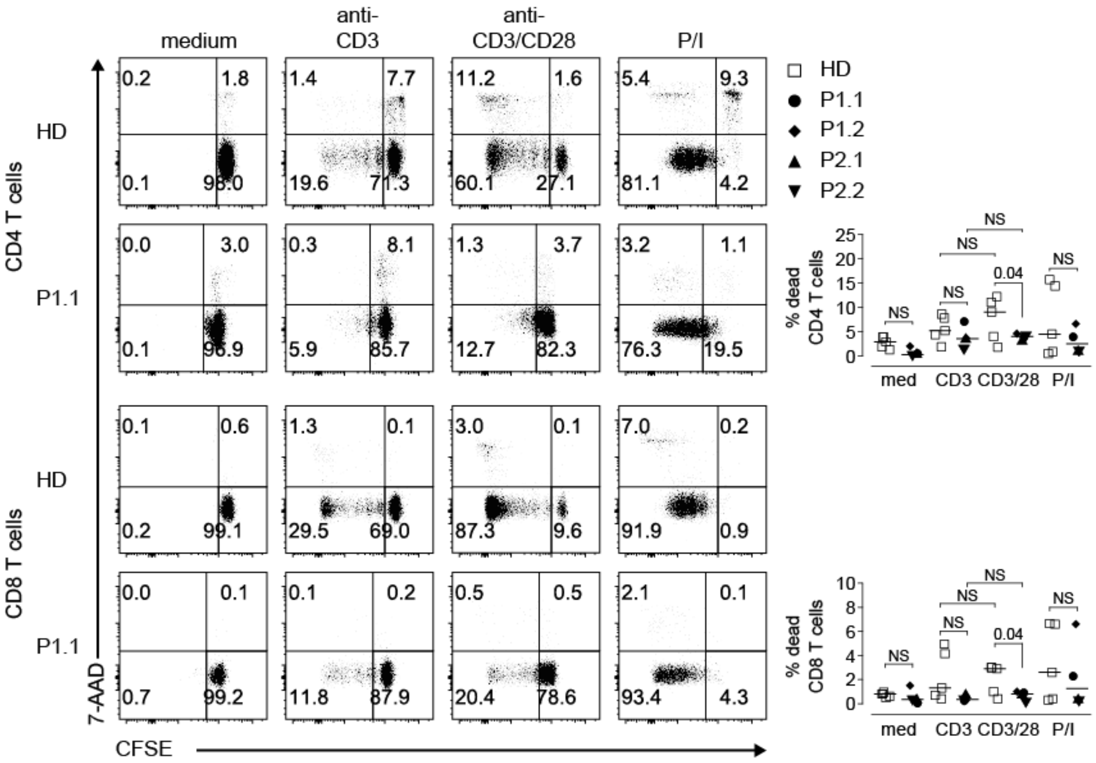


**Supplementary Figure 5.** CARMIL2-deficient T cells have reduced CD28-dependent activation-induced cell death. Dot plots of 7-AAD enrichment and CFSE dilution in CD4 and CD8 T cells left unstimulated (medium), or stimulated for five days with anti-CD3, anti-CD3/CD28 or PMA/ionomycin (P/I) and summary of CD4 and CD8 T cell death percentages for five HD (open squares), P1.1 (black circle), P1.2 (black rhomb), P2.1 (black up-pointing triangle) and P2.2 (black down-pointing triangle). Small horizontal lines indicate the median. Each symbol represents an individual donor. Data are representative for two independent experiments with n=2. Significance levels are calculated with Welch´s *t*-test and indicated in the summary graphs (NS = non significant).

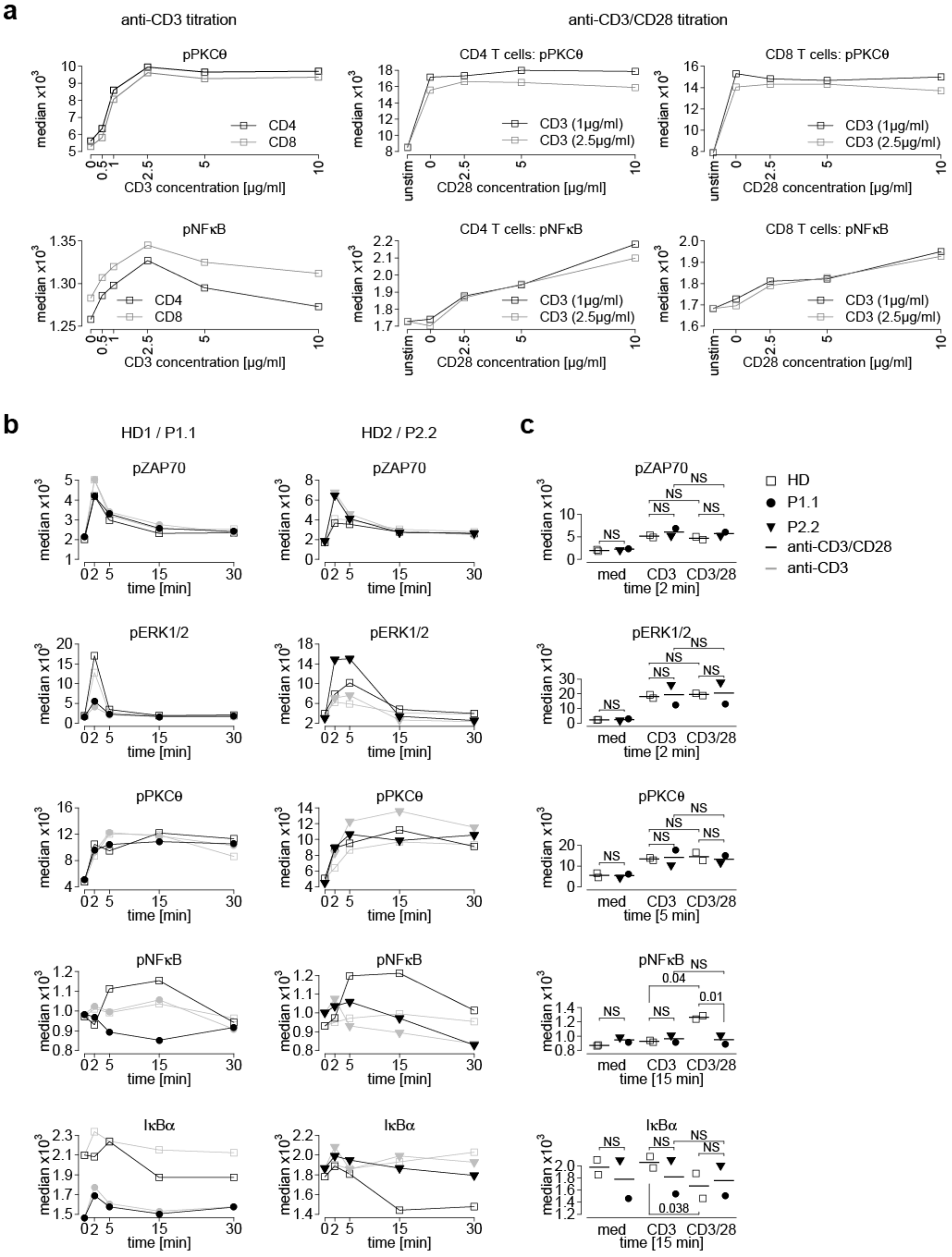


**Supplementary Figure 6.** CARMIL2-deficiency impairs CD28 co-signaling. (**a**) Stimulation titration curves with increasing anti-CD3 and anti-CD28 concetrations for pPKCθ and pNF-κB (p65) in CD4 and CD8 T lymphoblasts from HD. (**b**) Median of pZAP70, pERK1/2, pPKCθ, pNF-κB (p65) and IκBα in CD8 T lymphoblasts from HD1 and HD2 (open squares), P1.1 (black circle) and P2.2 (black down-pointing triangle) stimulated with anti-CD3 and anti-CD3/CD28 for 0, 2, 5, 15 and 30 min by flow cytometry. (**c**) Summary of median (phospho-) protein levels for two HD and two patients at the indicated time points with n=3. Significance levels are calculated with Welch´s *t*-test (NS = non significant).

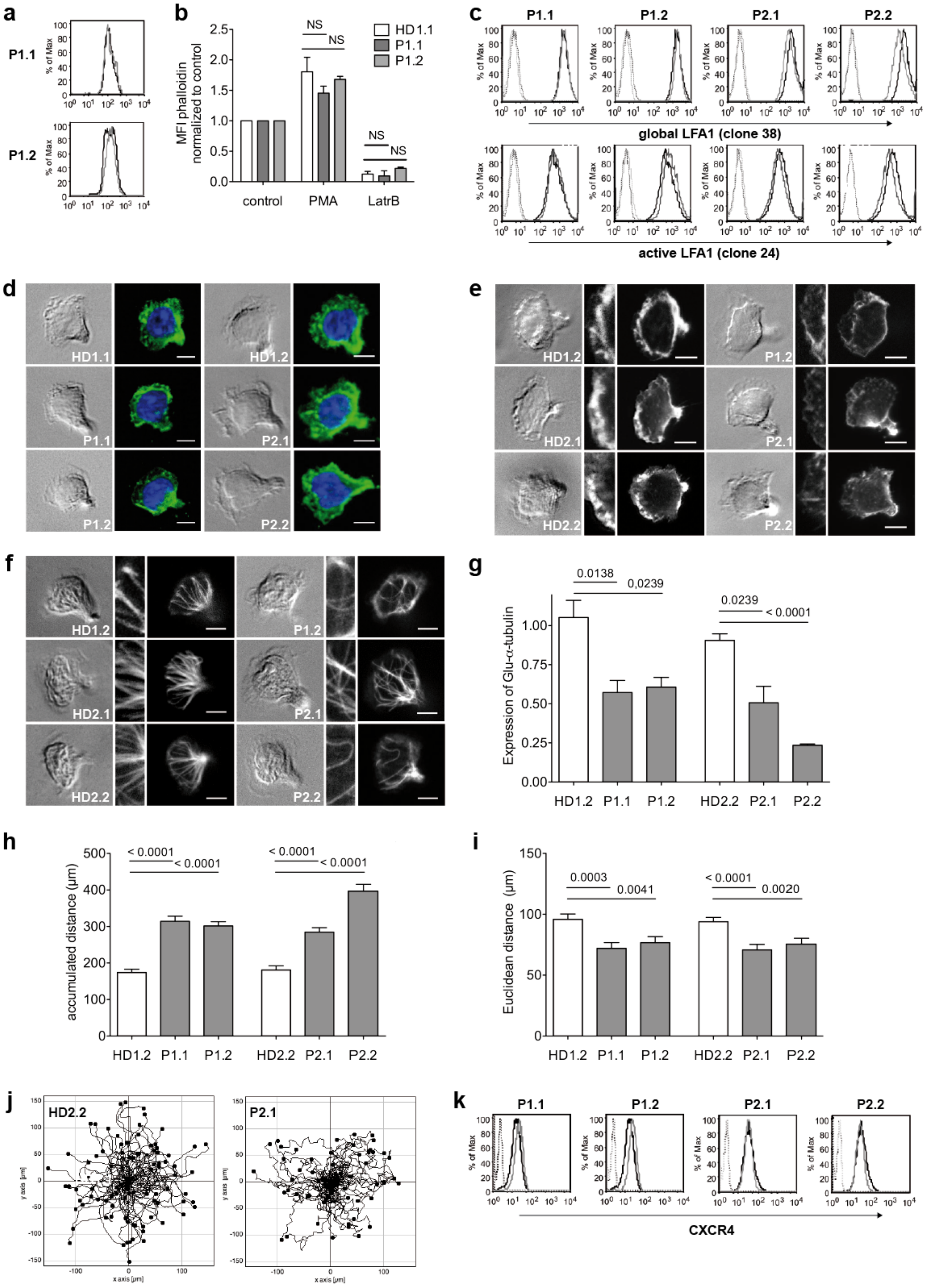


**Supplementary Figure 7.** CARMIL2-deficiency results in impaired cytoskeletal architecture and directness of migration. (**a**) F-actin amount detected by MFI of phalloidin-Alexa596

binding (black line, HD1.1; grey line, P1.1 or P1.2). (**b**) Quantification of F-actin in PMA or latrunculin B (LatrB) treated T lymphoblasts normalized to control cells of HD1.1, P1.1 and P1.2 (mean ± s.e.m., n=2). (**c**) Comparable amount of global LFA1 (clone 38) or active LFA1 (clone 24) in HD1.1 or HD2.1 compared to P1.1 and P1.2 or P2.1 and P2.2 T lymphoblasts, respectively. One representative histogram of two independent experiments is shown (black line, HD; grey line, patients; dotted lines, respective mAb controls). (**d**, **e**, **f**) DIC and fluorescence images of LFA1 (**d**, green; DAPI blue), F-actin (**e**) and tubulin (**f**) in T lymphoblasts from HD and patients migrating on ICAM1 (scale bars 5µm). Fluorescence images in (**d**) are maximum intensity projections of acquired z-stacks. Fluorescence pictures in (**e**, **f**) are single images at the level of the adhesion plane. Details of fluorescence images are shown in the middle. (**g**) Quantification of stable α-tubulin on immunoblots (Fig. 6c) as detected by Glu-α-tubulin normalized to the amount of Glu-α-tubulin in T lymphoblasts from HD1.1 (mean ± s.e.m., n≥3). (**h**, **i**, **j**) Spontaneous migration of HD and patient T lymphoblasts on ICAM1 in 2D and quantification of the accumulated distance (**h**, length of migration paths) and Euclidean distance (**i**, length of straight line between cell start and end point) (mean ± s.e.m.). (**j**) Paths of cell migration from HD2.2 and P2.1 are visualized on trajectory plots (n=99). (**k**) Comparable surface expression of the CXCL12 receptor CXCR4 detected by flow cytometry in HD1.1 or HD2.1 compared to P1.1 and P1.2 or P2.1 and P2.2, respectively. One representative histogram out of two independent experiments (black line, HD; grey line, patient T cells, dotted lines, respective mAb controls). Significance levels are calculated with the two-sided unpaired Student´s *t*-test and indicated in the summary graphs (NS = non significant).

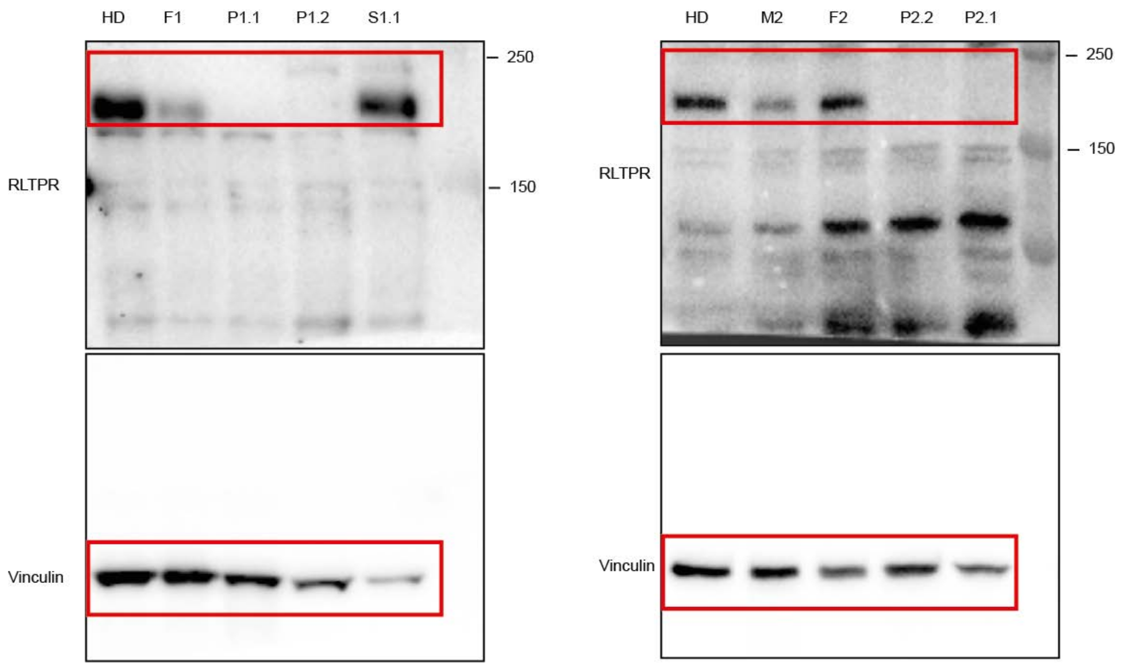


**Supplementary Figure 8.** Uncropped original immunoblots shown in Figure 2. The cropped areas are marked in red. Molecular weight markers are indicated in kDa.

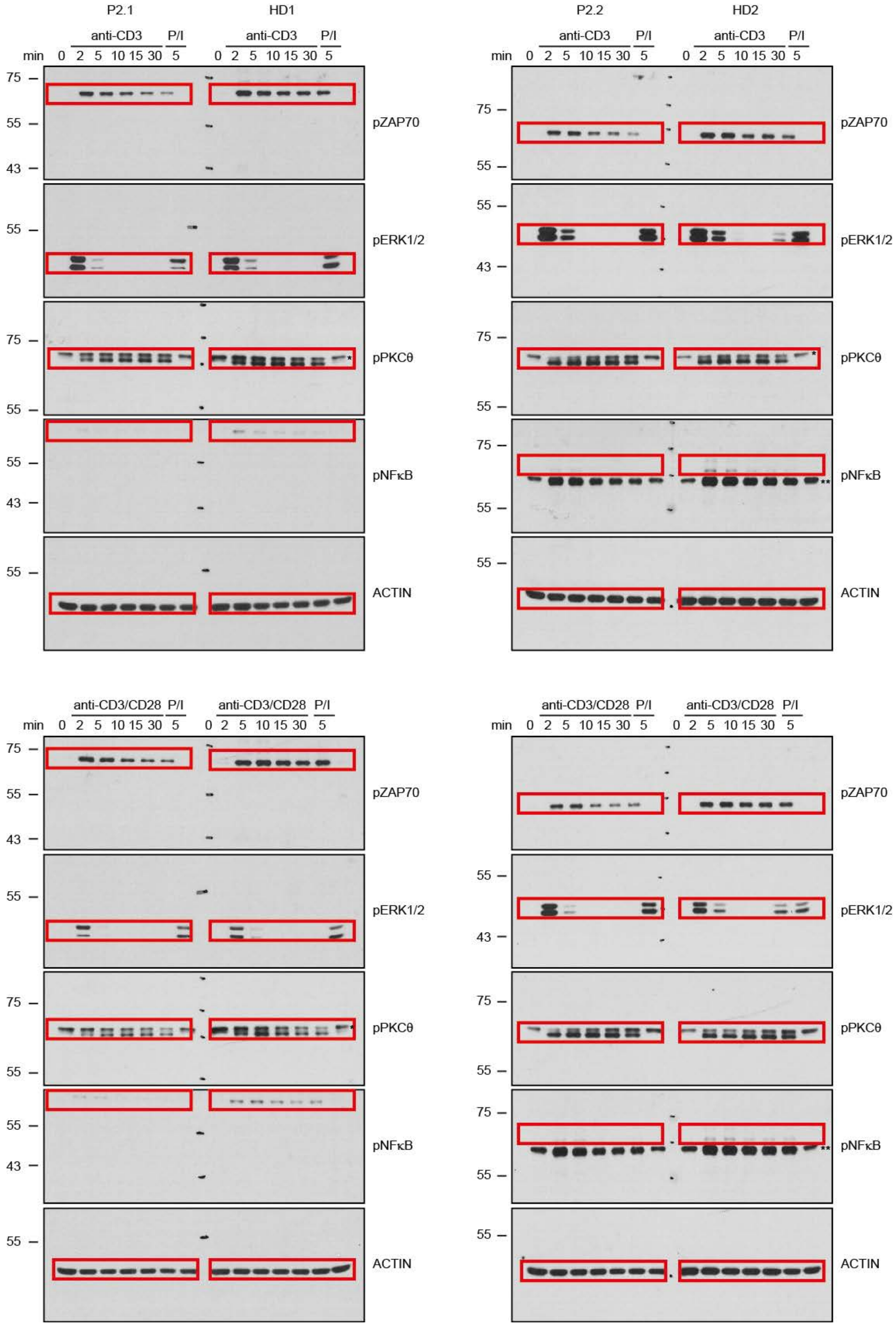


**Supplementary Figure 9.** Uncropped original immunoblots shown in Figure 7. The cropped areas are marked in red. Molecular weight markers are indicated in kDa. * non-specific band. ** blots were reprobed with pZAP70 antibody.

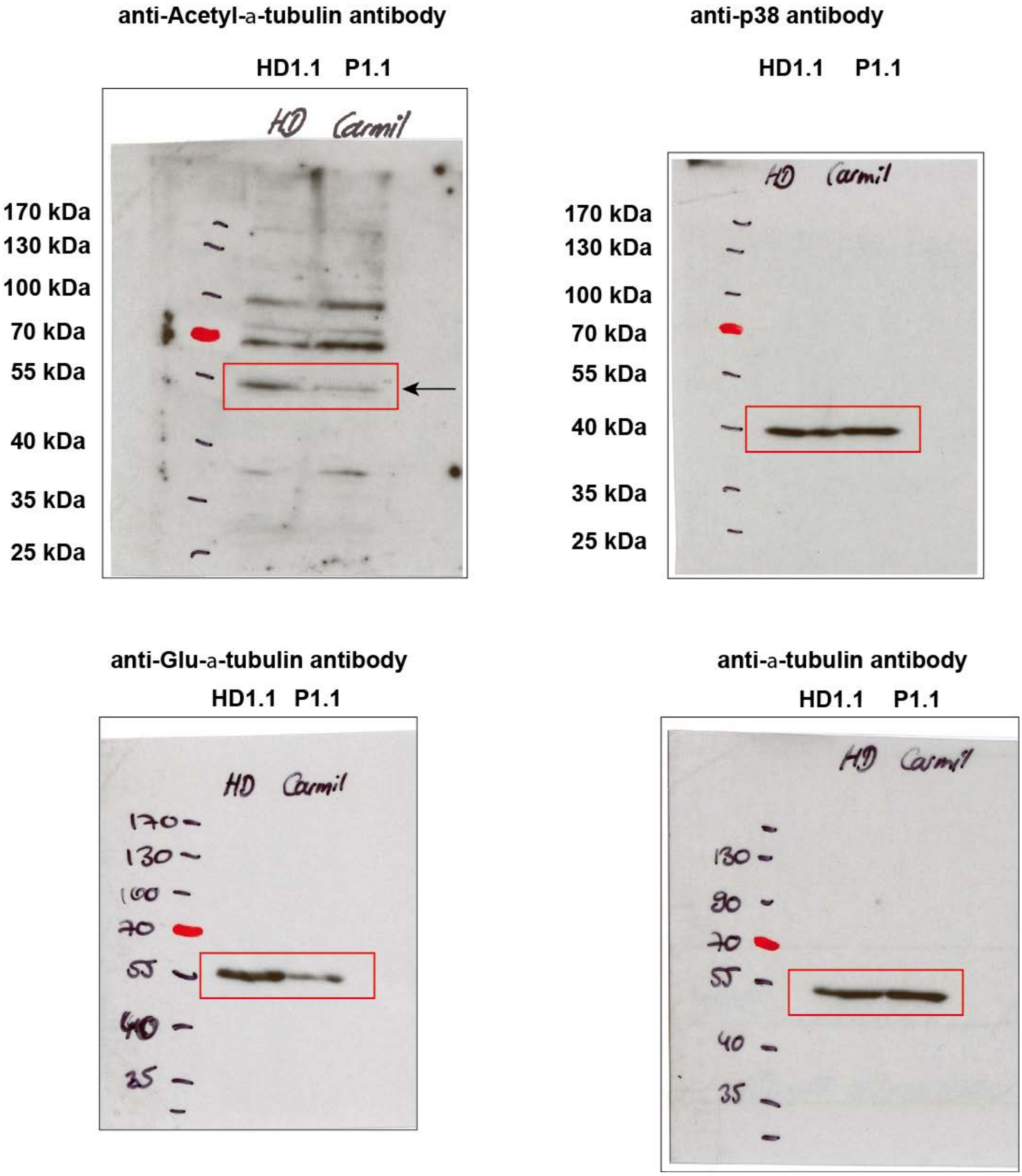


**Supplementary Figure 10.** Uncropped original immunoblots shown in Figure 8c. The cropped areas are marked in red. Molecular weight markers are indicated in kDa.

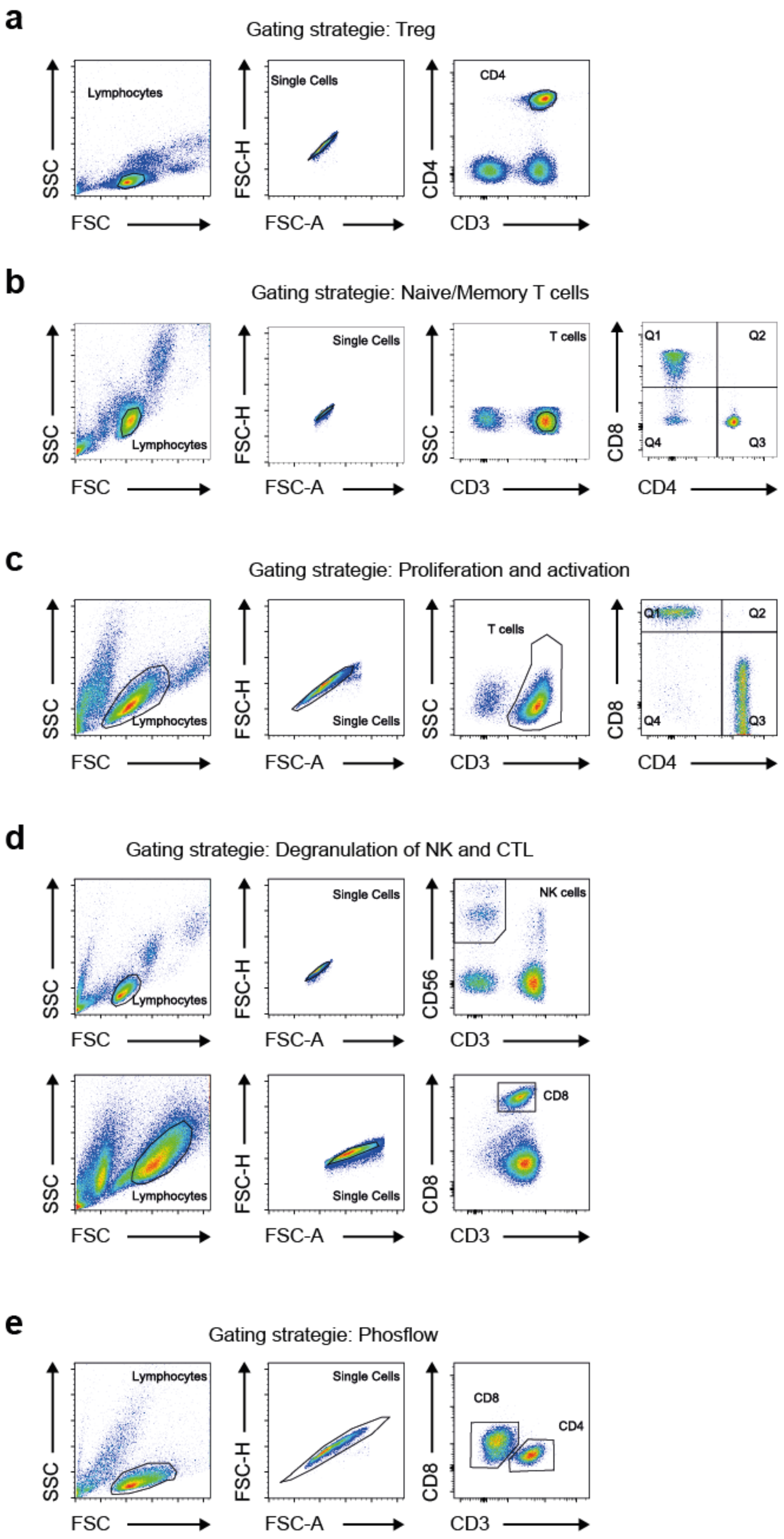


**Supplementary Figure 11.** The gating strategies used in Fig. 3a (**a**), in Fig. 3b and Supplementary Fig. 4a (**b**), in Fig. 4a, 4b and 5a and Supplementary Fig. 5(**c**), in Fig. 6a-c (**d**) and in Fig. 7b and Supplementary Fig. 6 (**e**).

**Supplementary Table 1.** Histopathological and histochemical characterization of $EBV^+$ SMT from P1.2.

| Marker | Result |
|---|---|
| Morphology | Spindle cells with fusiform or ovoid nuclei |
| Smooth muscle actin | Positive |
| Calponin | Positive |
| Pan-cytokeratin | Negative |
| Desmin | Negative |
| CD34 | Negative |
| C117 | Negative |
| S100 | Negative |
| Ki67 | Positive in 15-20% |
| EBER | Positive |
| LMP1 | Negative |
| LMP2A | Scattered positive |
| EBNA2 | Partially positive |
| ZEBRA | Negative |
| CD21 | Negative |
| P53 | Partial expression, no overexpression |
| C-MYC | Partial expression, no gene rearrangement |
| CD80/CD86 | Negative |

**Supplementary Tables 2.** Family 1 immune phenotype.

2a. EBV, CMV genome and antibody counts.

| **Family 1** | | | **P1.1** | **P1.2** |
|---|---|---|---|---|
| **Parameter*** | **Material** | **Method** | **Age 7y8m** | **Age 5y2m** |
| Epstein-Barr virus (EBV) | Serum | PCR# | negative | negative |
| Epstein-Barr virus (EBV) | Colon | PCR | 1,600 copies/ml | not done |
| Epstein-Barr virus (EBV) | Stool | PCR | not done | 350 copies/ml |
| Epstein-Barr virus (EBV) | Urine | PCR | not done | 900 copies/ml |
| Cytomegalovirus (CMV) | Urine | PCR | not done | 160,000 copies/ml |
| EBV-VCA IgM | Serum | EIA§ | 21 AU/ml (positive) | negative |
| EBV-VCA IgG | Serum | EIA | 26 AU/ml (positive) | 137 U/ml (positive)¶ |
| EBV-VCA IgA | Serum | IFT† | negative | not done |
| EBV-EA IgG | Serum | EIA | negative | negative¶ |
| EBV-EA IgA | Serum | IFT | negative | not done |
| EBV-EBNA IgG | Serum | EIA | negative | 5 U/ml (positive)¶ |
| CMV IgM | Serum | EIA | not done | 1:40 |
| CMV IgG | Serum | EIA | not done | 4.8 AU/ml¶ |

*Normal values are from the Max von Pettenkofer Institute, Ludwig-Maximilians-Universität, Munich, Germany; #PCR = polymerase chain reaction; §EIA = enzyme-linked immunosorbent assay; ¶P1.2 has had immunoglobulin replacement therapy a few months before that; †IFT = indirect immunofluorescence test.

2b. T, B and NK cell counts.

| **Family 1** | | | | **P1.1** | | | | **P1.2** | | | |
|---|---|---|---|---|---|---|---|---|---|---|---|
| | | **Normal value*** | | **Age 7y2m** | | **Age 7y3m** | | **Age 5y2m** | | **Age 5y6m** | |
| **Cell subset** | **Marker** | #/µl | % | #/µl | % | #/µl | % | #/µl | % | #/µl | % |
| T cells | $CD3^+$ per $CD45^+$ | 700-4200 | 55-78 | 3430 | 77 | 3935 | 79 | 5530 | 72 | 2474 | 89 |
| Helper T cells | $CD3^+CD4^+$ per $CD45^+$ | 300-2000 | 27-53 | 1960 | 44 | 2291 | 46 | 3380 | 44 | 1223 | 44 |
| Cytotoxic T cells | $CD3^+CD8^+$ per $CD45^+$ | 300-1800 | 19-34 | 1069 | 24 | 1196 | 24 | 1834 | 24 | 1001 | 36 |
| Activated T cells, B cells, monocytes | $HLA\text{-}DR^+$ per $CD45^+$ | 250-2300 | 13-45 | 1425 | 32 | 1295 | 32 | 2534 | 33 | 639 | 23 |
| Activated T cells | $CD3^+$ $HLA\text{-}DR^+$ per $CD45^+$ | 50-700 | 3-14 | 490 | 11 | 349 | 11 | 614 | 8 | 361 | 13 |
| B cells | $CD19^+$ per $CD45^+$ | 200-1600 | 10-31 | 891 | 20 | 847 | 20 | 1843 | 24 | 222 | 8 |
| NK cells | $CD3^-CD16^+CD56^+$ per $CD45^+$ | 90-900 | 4-26 | 134 | 3 | 150 | 3 | 154 | 2 | 56 | 2 |

*Normal values are from Comans-Bitter et al., J Pediatr 1997.

2c. B cell subset counts.

| Family 1 | | | | P1.1 | | P1.2 | |
|---|---|---|---|---|---|---|---|
| | | Normal value* | | Age 7y2m | | Age 5y2m | |
| **Cell subset** | **Marker** | #/µl | % | #/µl | % | #/µl | % |
| B cells | $CD19^+$ per lymphocyte gate | 92-792 | 4.3-23.1 | 615 | 13.8 | 1244 | 16.2 |
| Memory B cells | $CD19^+CD27^+$ | 9-136 | 4.6-49.1 | 32 | 5.2 | 69 | 5.6 |
| IgM memory B cells | $CD19^+CD27^+IgM^+IgD^+$ | 1-43 | 0.2-12.3 | 25 | 4.0 | 51 | 4.1 |
| Switched memory B cells | $CD19^+CD27^+IgM^-IgD^-$ | 5-74 | 1.9-30.4 | 6 | 1.0 | 15 | 1.2 |
| $CD21^{low}CD38^{low}$ B cells | $CD19^+CD21^{low}CD38^{low}$ | 1-26 | 0.2-5.5 | 13 | 2.1 | 37 | 3 |

*Normal values are from Mayo Medical Laboratories, Test ID: IABCS.

2d. Immunoglobulin and specific antibody counts.

| Family 1 | P1.1* | | P1.2* | |
|---|---|---|---|---|
| | **Normal value#** | **Age 7y2m** | **Normal value** | **Age 5y2m** |
| Total protein | 6.0-8.0 g/l | 8.0 g/l | 6.0-8.0 g/l | 6.8 g/dl |
| IgM | 0.48-2.38 g/l | 1.97 g/l | 0.48-2.38 g/l | 2.05 g/l |
| IgG | 5.76-15.1 g/l | 8.91 g/l | 5.76-15.1 g/l | 4.62 g/l§ |
| $IgG_1$ | 3.50-9.10 g/l | 4.87 g/l | 3.00-8.40 g/l | 2.38 g/l§ |
| $IgG_2$ | 0.85-3.30 g/l | 2.44 g/l | 0.70-2.55 g/l | 1.75 g/l§ |
| $IgG_3$ | 0.20-1.04 g/l | 0.57 g/l | 0.17-0.97 g/l | 0.31 g/l§ |
| $IgG_4$ | 0.03-1.58 g/l | 0.01 g/l | 0.02-1.16 g/l | <0.01 g/l§ |
| IgA | 0.46-3.04 g/l | 1.23 g/l | 0.46-3.04 g/l | 1.88 g/l |
| IgE | ≤90 IU/ml | 17.6 IU/ml | ≤90 IU/ml | 0.9 IU/ml |
| Tetanus toxoid IgG | ≥0.1 IU/ml | 0.09 IU/ml | ≥0.1 IU/ml | 0.15 IU/ml§ |
| Diphtheria toxoid IgG | ≥0.1 IU/ml | 0.07 IU/ml | ≥0.1 IU/ml | 0.04 IU/ml§ |
| Haemophilus influenzae typ b IgG | ≥0.15 mg/l | <0.1 mg/l | ≥0.15 mg/l | 0.15 mg/l§ |
| Pneumococcal polysaccharide IgG | ≥21.6 mg/l | 49.2 mg/l | ≥21.6 mg/l | 6.8 mg/l§ |

*P1.1 and P1.2 vaccination schedule: not documented, but reported to be in accordance with the vaccination schedule recommended in Yemen;
#Normal values are from and Baudner and Dati, J Lab Med 1996; Schauer et al., Clin Chem 2003; Schröder et al., Dtsch Med Wochenschr 1992; Efstratiou et al., Comm Dis Public Health 1999; Schauer et al., Clin Diagn Lab Immunol 2003;
§P1.2 has received immunoglobulin substitution several months before.

2e. T and B cell proliferation indices.

| **Family 1** | **P1.1*** | | **P1.2*** | |
|---|---|---|---|---|
| | **Normal value#** | **Age 7y2m** | **Normal value** | **Age 5y2m** |
| No stimulation | ≥ 0.15 | 0.91 | ≥ 0.15 | 1.06 |
| PHA§ 1 µg/ml | ≥ 0.15 | 1.48 | ≥ 0.15 | 0.46 |
| PHA 0.1 µg/ml | ≥ 0.15 | 1.46 | ≥ 0.15 | 0.07 |
| OKT3† 10 ng/ml | ≥ 0.15 | 0.55 | ≥ 0.15 | 0.69 |
| OKT3 1 ng/ml | ≥ 0.15 | 0.25 | ≥ 0.15 | 0.41 |
| SAC¶ 0.001% | ≥ 0.15 | 0.15 | ≥ 0.15 | 0.17 |
| Antigen° 0.5/1.3 Lf/ml | ≥ 0.15 | 0.03 | ≥ 0.15 | 0.02 |

*P2.1 vaccination schedule: not documented, but reported to be in accordance with the vaccination schedule recommended in Yemen;
#Normal values are in house derived;
§PHA = phytohemagglutinin;
†OKT3 = monoclonal anti-CD3ε antibody;
¶SAC = Staphylococcus aureus Cowan strain I;
°Antigen = tetanus and diphtheria toxoid.

**Supplementary Tables 3.** Family 2 immune phenotype.

3a. EBV, CMV, HHV6 genome and antibody counts.

| **Family 2** | | | **P2.1** | **P2.2** |
|---|---|---|---|---|
| **Parameter*** | **Material** | **Method** | **Age 14y1m** | **Age 11y10m** |
| Epstein-Barr virus (EBV) | Serum | PCR[#] | 330,000 copies/ml | negative |
| Cytomegalovirus (CMV) | Serum | PCR | 70,000 copies/ml | negative |
| Human herpesvirus 6 (HHV6) | Serum | PCR | 8,800 copies/ml | negative |
| EBV-VCA IgM | Serum | EIA[§] | 13 AU/ml (positive) | negative |
| EBV-VCA IgG | Serum | EIA | >200 U/ml (positive) | 143 U/ml (positive) |
| EBV-VCA IgA | Serum | IFT† | 1:128 (positive) | 1:64 (positive) |
| EBV-EA IgG | Serum | EIA | negative | negative |
| EBV-EA IgA | Serum | IFT | negative | negative |
| EBV-EBNA IgG | Serum | EIA | negative | negative |
| CMV IgM | Serum | EIA | positive | negative |
| CMV IgG | Serum | EIA | 14 AU/ml (positive) | negative |

*Normal values are from the Max von Pettenkofer Institute, Ludwig-Maximilians-Universität, Munich, Germany; #PCR = polymerase chain reaction; §EIA = enzyme-linked immunosorbent assay; †IFT = indirect immunofluorescence test.

3b. T, B and NK cell counts.

| **Family 2** | | | | **P1.1** | | **P1.2** | |
|---|---|---|---|---|---|---|---|
| | | **Normal value*** | | **Age 13 y** | | **Age 11 y** | |
| **Cell subset** | **Marker** | #/µl | % | #/µl | % | #/µl | % |
| T cells | $CD3^+$ per $CD45^+$ | 800-3500 | 52-78 | 3655 | 93 | 3377 | 86 |
| Helper T cells | $CD3^+CD4^+$ per $CD45^+$ | 400-2100 | 25-48 | 1847 | 47 | 1964 | 50 |
| Cytotoxic T cells | $CD3^+CD8^+$ per $CD45^+$ | 200-1200 | 9-35 | 1533 | 39 | 1178 | 30 |
| Activated T cells, B cells, monocytes | $HLA\text{-}DR^+$ per $CD45^+$ | 220-800 | 9-32 | 590 | 15 | 589 | 15 |
| Activated T cells | $CD3^+$ $HLA\text{-}DR^+$ per $CD45^+$ | 20-200 | 1-8 | 393 | 10 | 118 | 3 |
| B cells | $CD19^+$ per $CD45^+$ | 200-600 | 8-24 | 157 | 4 | 471 | 12 |
| NK cells | $CD3^-CD16^+CD56^+$ per $CD45^+$ | 70-1200 | 6-27 | 39 | 1 | 79 | 2 |

*Normal values are from Comans-Bitter et al., J Pediatr 1997.

3c. B cell subset counts.

| Family 2 | | | | P2.1 | | P2.2 | |
|---|---|---|---|---|---|---|---|
| | | Normal value* | | Age 13y4m | | Age 11y1m | |
| **Cell subset** | **Marker** | #/µl | % | #/µl | % | #/µl | % |
| B cells | $CD19^+$ per lymphocyte gate | 92-792 | 4.3-23.1 | 110 | 2.8 | 192 | 4.9 |
| Memory B cells | $CD19^+CD27^+$ | 9-136 | 4.6-49.1 | 14 | 12.4 | 10 | 5.4 |
| IgM memory B cells | $CD19^+CD27^+IgM^+IgD^+$ | 1-43 | 0.2-12.3 | 6 | 5.9 | 7 | 3.4 |
| Switched memory B cells | $CD19^+CD27^+IgM^-IgD^-$ | 5-74 | 1.9-30.4 | 6 | 5.9 | 3 | 1.5 |
| $CD21^{low}CD38^{low}$ B cells | $CD19^+CD21^{low}CD38^{low}$ | 1-26 | 0.2-5.5 | 6 | 5.4 | 4 | 2.3 |

*Normal values are from Mayo Medical Laboratories, Test ID: IABCS.

3d. Recent thymic emigrant cell counts.

| Family 2 | | | | P2.1 | | P2.2 | |
|---|---|---|---|---|---|---|---|
| | | Normal value* | | Age 13y4m | | Age 11y1m | |
| **Cell subset** | **Marker** | #/µl | % | #/µl | % | #/µl | % |
| T cells | $CD3^+$ per lymphocyte gate | 800-3500 | 52-78 | 3270 | 83.2 | 3389 | 86.3 |
| RTE cells | $CD4^+CD31^+CD45RA^+$ per $CD3^+$ | 50-926 | 19.4-60.9 | 1001 | 62.1 | 1334 | 66.6 |

*Normal values are from Mayo Medical Laboratories, Test ID: 89504.

3e. CD45RA/CD45R0 T cell counts.

| Family 2 | | | | P2.1 | | P2.2 | |
|---|---|---|---|---|---|---|---|
| | | Normal value* | | Age 13y4m | | Age 11y1m | |
| **Cell subset** | **Marker** | #/µl | % | #/µl | % | #/µl | % |
| T cells | $CD3^+$ per lymphocyte gate | 800-3500 | 52-78 | 3262 | 83 | 3377 | 86 |
| Helper T cells | $CD4^+$ per $CD3^+$ | 400-2100 | 25-48 | 1611 | 41 | 2003 | **51** |
| CD45RA helper T cells | $CD4^+CD45RA^+$ per $CD4^+$ | 135-893 | 21-75 | 1402 | 87 | 1742 | 87 |
| CD45R0 helper T cells | $CD4^+CD45R0^+$ per $CD4^+$ | 56-411 | 11-44 | 209 | 13 | 260 | 13 |

*Normal values are from Mayo Medical Laboratories, Test ID: TCP.

3f. Αβ/γδ T cell counts.

| Family 2 | | | | P2.1 | | P2.2 | |
|---|---|---|---|---|---|---|---|
| | | Normal value[*] | | Age 13y4m | | Age 11y1m | |
| **Cell subset** | **Marker** | #/µl | % | #/µl | % | #/µl | % |
| T cells | CD3$^+$ per lymphocyte gate | 800-3500 | 52-78 | 3270 | 83.2 | 3389 | 86.3 |
| Αβ DN[#] T cells | TCRαβ$^+$CD4$^-$CD8$^-$ per CD3$^+$ | <35 | <2 | 23 | 0.71 | 20 | 0.58 |
| Γδ DN T cells | TCRγδ$^+$CD4$^-$CD8$^-$ per CD3$^+$ | n.a.† | 3-10 | 217 | 6.64 | 221 | 6.52 |
| Γδ SP[§] T cells | TCRγδ$^+$CD4$^-$CD8$^+$ per CD3$^+$ | n.a. | n.a. | 163 | 4.99 | 112 | 3.31 |

[*]Normal values are from Mayo Medical Laboratories, Test ID: 82449;
[#]DN = double negative;
[§]SP = single positive;
†n.a. = not available.

3g. Immunoglobulin and specific antibody counts/vaccination responses.

| Family 2 | P2.1[*] | | P2.2[#] | |
|---|---|---|---|---|
| | **Normal value[§]** | **Age 13y4m** | **Normal value** | **Age 11y1m** |
| Total protein | 6.0-8.0 g/dl | 6.3 g/dl | 6.0-8.0 g/dl | 7.5 g/dl |
| IgM | 0.4-2.3 g/l | 1.6 g/l | 0.4-2.3 g/l | 2.1 g/l |
| IgG | 7.0.-16.0 g/l | 11.3 g/l | 7.0.-16.0 g/l | 10.5 g/l |
| $IgG_1$ | 3.7-9.1 g/l | 4.75 g/l | 3.7-9.3 g/l | 6.32 g/l |
| $IgG_2$ | 1.10-4.85 g/l | 5.41 g/l | 1.0-4.0 g/l | 3.10 g/l |
| $IgG_3$ | 0.24-1.16 g/l | 0.67 g/l | 0.22-1.09 g/l | 0.37 g/l |
| $IgG_4$ | 0.052-1.961 g/l | 0.179 g/l | 0.043-1.9 g/l | 0.133 g/l |
| IgA | 0.7-4.0 g/l | 3.5 g/l | 0.7-4.0 g/l | 2.6 g/l |
| IgE | ≤200 IU/ml | 75IU/ml | ≤200 IU/ml | 758 IU/ml |
| Tetanus toxoid IgG | ≥0.1 IU/ml | 0.02 IU/ml | ≥0.1 IU/ml | <0.01 IU/ml |
| Diphtheria toxoid IgG | ≥0.1 IU/ml | <0.01 IU/ml | ≥0.1 IU/ml | <0.01 IU/ml |
| Haemophilus influenzae typ b IgG | ≥0.15 mg/l | 0.16mg/l | ≥0.15 mg/l | 0.78mg/l |
| Pneumococcal polysaccharide IgG | ≥21.6 mg/l | 38.1mg/l | ≥21.6 mg/l | 19.7 mg/l |
| Bacille Calmette-Guérin | scar | no scar | scar | no scar |

[*]P2.1 vaccination schedule: 1x BCG, 4x oPV, 4x DPT, 4x Hib, 4x HB, 2x MMR, 1x YF 1x, 2x IN;
[#]P2.2 vaccination schedule: 1x BCG, 4x oPV, 4x DPT, 4x Hib, 4x HB, 1x MMR, 1x YF 1x, 2x IN;
[§]Normal values are from and Baudner and Dati, J Lab Med 1996; Schauer et al., Clin Chem 2003; Schröder et al., Dtsch Med Wochenschr 1992; Efstratiou et al., Comm Dis Public Health 1999; Schauer et al., Clin Diagn Lab Immunol 2003;

**Supplementary Table 4.** WES candidate variants for family 1 after filtering.

| Position | Orig Var | RSID | EnsGene | HGNC ID | EnsTrans | Type | Variation | PolyPhen | SIFT | ROH* |
|---|---|---|---|---|---|---|---|---|---|---|
| chr16:67680638-67680638 | A->AG | none | ENSG00000159753 | CARMIL2 | ENST00000334583 | frame-shift | 489+G,*@167 | n.a. | n.a. | yes |
| chr16:72992196-72992196 | C->T | 111320371 | ENSG00000140836 | ZFHX3 | ENST00000268489 | missense | 1849G>A,617V>I | 0.105 | n.a. | yes |
| chr16:78458922-78458922 | G->A | none | ENSG00000186153 | WWOX | ENST00000566780 | missense | 761G>A,254R>H | 1 | 0 | yes |
| chr20:39792419-39792419 | C->T | 150381500 | ENSG00000124181 | PLCG1 | ENST00000373271 | missense | 956C>T,319P>L | 0.029 | n.a. | no |
| chr16:72057118-72057118 | C->T | none | ENSG00000102967 | DHODH | ENST00000219240 | missense | 874C>T,292L>F | 0.992 | 0 | yes |
| chr16:82131973-82131973 | C->A | none | ENSG00000086696 | HSD17B2 | ENST00000199936 | missense | 1096C>A,366H>N | 0 | 0.37 | yes |

*ROH = candidate identified by WES located inside the regions identified by homozygosity mapping.

**Supplementary Table 5.** Primers used for Sanger sequencing.

| Gene | Type of DNA | Mutation | Direction | Primer |
|---|---|---|---|---|
| *CARMIL2* | gDNA | c.489insG | Forward | 5’ GCCCCAAACTGAACAGAGCCATT 3’ |
| *CARMIL2* | gDNA | c.489insG | Reverse | 5’ TCCTGGGTGAGAAGAAGATGCTTA 3’ |
| *CARMIL2* | gDNA | c.871+1G>T | Forward | 5’ AGAGGAGAGTCTGGAGGCTTG 3‘ |
| *CARMIL2* | gDNA | c.871+1G>T | Reverse | 5’ CACTCAGCAGTTGGACAGACC 3‘ |
| *CARMIL2* | cDNA | c.871+1G>T | Forward | 5’ CCTTGAGGTCTCAGAACAGATTCTGC 3’ |
| *CARMIL2* | cDNA | c.871+1G>T | Reverse | 5’ CTGCGAGATTCAGGAACGACAGTA 3’ |
